# Dealing with indeterminate outcomes in antimalarial drug efficacy trials: a comparison between complete case analysis, multiple imputation and inverse probability weighting

**DOI:** 10.1186/s12874-019-0856-z

**Published:** 2019-11-27

**Authors:** Prabin Dahal, Kasia Stepniewska, Philippe J. Guerin, Umberto D’Alessandro, Ric N. Price, Julie A. Simpson

**Affiliations:** 1 WorldWide Antimalarial Resistance Network (WWARN), Oxford, UK; 2 Centre for Tropical Medicine and Global Health, Nuffield Department of Clinical Medicine, University of Oxford, Oxford, UK; 3 Medical Research Council Unit, Fajara, The Gambia; 4 Unit of Malariology, Institute of Tropical Medicine, Antwerp, Belgium; 5 Global and Tropical Health Division, Menzies School of Health Research and Charles Darwin University, Darwin, Australia; 6 Centre for Epidemiology and Biostatistics, Melbourne School of Population and Global Health, The University of Melbourne, Melbourne, Australia

**Keywords:** *Plasmodium falciparum*, Efficacy, Indeterminate outcomes, Multiple imputation, Inverse probability weighting

## Abstract

**Background:**

Antimalarial clinical efficacy studies for uncomplicated *Plasmodium falciparum* malaria frequently encounter situations in which molecular genotyping is unable to discriminate between parasitic recurrence, either new infection or recrudescence. The current WHO guideline recommends excluding these individuals with indeterminate outcomes in a complete case (CC) analysis. Data from the four artemisinin-based combination (4ABC) trial was used to compare the performance of multiple imputation (MI) and inverse probability weighting (IPW) against the standard CC analysis for dealing with indeterminate recurrences.

**Methods:**

3369 study participants from the multicentre study (4ABC trial) with molecularly defined parasitic recurrence treated with three artemisinin-based combination therapies were used to represent a complete dataset. A set proportion of recurrent infections (10, 30 and 45%) were reclassified as missing using two mechanisms: a completely random selection (mechanism 1); missingness weakly dependent (mechanism 2a) and strongly dependent (mechanism 2b) on treatment and transmission intensity. The performance of MI, IPW and CC approaches in estimating the Kaplan-Meier (K-M) probability of parasitic recrudescence at day 28 was then compared. In addition, the maximum likelihood estimate of the cured proportion was presented for further comparison (analytical solution). Performance measures (bias, relative bias, standard error and coverage) were reported as an average from 1000 simulation runs.

**Results:**

The CC analyses resulted in absolute underestimation of K-M probability of day 28 recrudescence by up to 1.7% and were associated with reduced precision and poor coverage across all the scenarios studied. Both MI and IPW method performed better (greater consistency and greater efficiency) compared to CC analysis. In the absence of censoring, the analytical solution provided the most consistent and accurate estimate of cured proportion compared to the CC analyses.

**Conclusions:**

The widely used CC approach underestimates antimalarial failure; IPW and MI procedures provided efficient and consistent estimates and should be considered when reporting the results of antimalarial clinical trials, especially in areas of high transmission, where the proportion of indeterminate outcomes could be large. The analytical solution estimating the cured proportion could provide an alternative approach, in scenarios with minimal censoring due to loss to follow-up or new infections.

## Background

The primary endpoint in efficacy studies for antimalarials in uncomplicated *Plasmodium falciparum* malaria is the risk of recrudescence, defined as the recurrence of peripheral parasitaemia genetically identical to the parasites present before treatment. Molecular analysis of the parasite samples collected at pre-treatment and on the day of recurrence is used to discriminate homologous (recrudescent) from heterologous (new) infections [1]. When paired analysis of the pre- and post-treatment parasite cannot be determined reliably, treatment outcome is defined as indeterminate (Additional file 1, Section A).

The current WHO guideline for dealing with indeterminate outcomes in antimalarial efficacy trials is to exclude them from the analysis, that is, to carry out a complete case (CC) analysis [2]. This implicitly assumes that the indeterminate cases are a representative random sample of the study population, ignoring the fact that these indeterminate recurrences must be either a recrudescence or new infection, and may depend on other measured and unmeasured patient and parasite characteristics. The CC analysis is usually supplemented with two extreme sensitivity analyses representing the worst and best scenarios, where all indeterminate recurrences are assumed to be either recrudescences or new infections. As well as biased, such ad hoc single imputation approaches consider the imputed datum as the ‘known observed’ value and uncertainty regarding not knowing the reason for parasite recurrence isn’t fully accounted for. This yields inferences that are over-precise, i.e. standard errors are too small rendering the associated hypothesis tests as invalid [3–5].

Under the multinomial assumption, the maximum likelihood estimate of the proportion of patients with parasitic recrudescence can be obtained as outlined by Little and Rubin (2002) [6]. Let, *n* be the total number of patients who received antimalarial drug, of whom *n*_0_ were cured, *m*_1_ developed new infection, *m*_2_ were recrudescent, and *r* recurrences were indeterminate at the end of the planned follow-up. The maximum likelihood estimate of proportion of who failed is then obtained as:

1 $$ {\hat{\rho}}_{ML}=\left(\frac{m_2}{m_1+{m}_2}\right).\left(\frac{n-{n}_0}{n}\right) $$

The complement of equation (1) provides an estimate of the cured proportion:

2 $$ 1-{\hat{\rho}}_{ML}=1-\left\{\left(\frac{m_2}{m_1+{m}_2}\right).\left(\frac{n-{n}_0}{n}\right)\right\} $$

In the absence of censoring, equation 2 provides a consistent estimate of failure proportion compared to the CC approach (Additional file 1, Section B). When there are censored observations (e.g: due to lost to follow-up or due to new infection), the Kaplan-Meier (K-M) method can be used. The K-M approach is currently the WHO recommended approach for measuring antimalarial failure, whereby individuals with indeterminate parasite recurrence are excluded and individuals with new infections or loss to follow-up are censored [2].

Alternative approaches for dealing with an indeterminant parasite recurrence outcome are multiple imputation (MI) and inverse probability weighting (IPW), which are statistically principled approaches for handling missing data [7–11] under the assumption that the missing data depends on observed variables. In antimalarial clinical efficacy studies, variables that are commonly recorded and may affect whether or not a recurrence is indeterminant, are transmission intensity, the number of molecular markers used, density of the parasites on day of recurrence and antimalarial treatment administered. Background allelic diversity of the parasite strain is rarely known or reported and therefore it is not possible to test if this variable influences the determination of homologous (recrudescence) or heterologous (new infection) parasite recurrences. As such, MI and IPW assume that the occurrence of indeterminant recurrences does not depend on allelic diversity of the parasite strain and any other unmeasured variables.

The basic principle of MI is to impute the missing values based on the distribution of the observed data and repeat this *m* times in order to account for the uncertainty in missing values [12, 13]. This is essentially a two-step procedure. In the first step, incomplete data are replicated multiple times from a suitable imputation model where values are drawn from the posterior predictive distribution (imputation step) [14]. In the second (analysis) step, the substantive model (target analysis) of interest is carried out on each of the completed datasets (observed plus imputed data). The final estimates and standard errors are then derived by combining estimates across each of the multiply imputed datasets using Rubin’s combination rules, which incorporates uncertainties within and between imputations [13]. For IPW, complete cases are weighted by the inverse of their probability of being a complete case, i.e. up-weighting the data from participants who have a low probability of being observed thus creating a pseudo-population [9]. The final analysis is then carried out using only the complete observations (i.e. for this example indeterminate recurrences are not included), but they are now weighted to rebalance the set of complete cases so that it is representative of the whole sample. Like MI, the IPW approach is also a two-step estimator. In the first step, a missingness model is constructed to estimate the probability of an observation being a complete case and the inverse of these probabilities are used as the weights in the analysis (step 2) of the complete cases.

Multiple Imputation and inverse probability weighting has been increasingly used in the medical and statistical literature in the past decade [9, 10, 15]. Yet only a handful of studies have considered these missing data methods when dealing with indeterminate outcomes in derivation of antimalarial efficacy in uncomplicated *P. falciparum* malaria (only three studies to our knowledge) [16–18]. Machekano et al. (2008) compared the performance of MI and IPW approaches against the CC analysis using a randomised study in Uganda in estimating drug efficacy using proportions [16]. Mukaka et al. (2016) compared MI against CC when estimating the risk difference between two antimalarial regimens [17]. In the PREGACT study (2017), MI was used as a sensitivity analysis to assess the robustness of the derived estimate of cured proportion [18]. None of the studies to date have compared the utility of MI and IPW approaches in handling indeterminate outcomes when deriving Kaplan-Meier (K-M) ($\hat{S_{KM}}$) estimates of drug efficacy for antimalarial regimens.

The aim of this simulation study was to assess the performance of MI and IPW approaches for handling indeterminate recurrences when estimating the day 28 proportion of parasitic recrudescence following antimalarial treatment using K-M survival analysis against those derived using the widely used CC approach. Specifically, the evaluation is based on a large multi-centre trial of four artemisinin-based combination therapies (4ABC trial) [19] in which we redraw and assign a set proportion (10, 30 and 45%) of known recurrences (recrudescences and new infections) to indeterminate (i.e. missing).

## Methods

### Motivating study

The four artemisinin-based combinations (4ABC) trial was a large multi-centric study (4116 patients enrolled) conducted at 12 sites in seven sub-Saharan African countries between 2007 to 2009 [19]. Four regimens were assessed: artemether-lumefantrine (AL), artesunate-amodiaquine (ASAQ), dihydroartemisinin-piperaquine (DP) and chlorproguanil-dapsone-artesunate (CDA). Patients were followed actively for up to 28 days. CDA was discontinued partway through the study due to reports from another phase III study of severe haemolysis. For this reason, data from only the AL, ASAQ and DP arms were considered in this simulation (*n* = 3431, Table 1). The trial is one of the largest antimalarial studies ever conducted and well suited to study the utility of MI and IPW approaches for handling indeterminate recurrences. The primary analysis of the 4ABC trial was the estimation of antimalarial drug efficacy at day 28, using the Kaplan-Meier (K-M) $\left({\hat{S}}_{KM}\right)$ method for each of the treatment regimens.

In total there were 62 (1.8%) indeterminate outcomes in the motivational study which were excluded and the remaining data (3369 observations) with known outcomes (81 recrudescence, 455 new infection, and 2833 who reached the planned end of the study (i.e. day 28) without observing any recurrence) were considered as a complete dataset for the purpose of this simulation study (referred to **full data** here onwards). The K-M estimates and associated standard errors (SEs) and the estimates of the cured proportions (SEs) estimated from the “full data” before inducing missingness are presented in Table 2 and referred to as the **full data estimate** hereafter. In the derivation of the K-M estimates, new infections were censored on the day of recurrence whereas they were considered as treatment success when deriving the cured proportion as recommended by the WHO [2]. The former estimates were used for evaluating the performance measures of the CC, IPW and MI approaches for estimating probability of day 28 cure whereas the latter estimates were used for evaluating the performance measures of the analytical solution (equation 2).

**Table 1:** Antimalarial treatment outcomes for the 4ABC Trial [19]

Treatment	Cured	Missing outcome (indeterminate recurrence)	Recrudescence	New Infection	Total
AL	847 (73.0%) †	29 (2.5%)	41 (3.5%)	243 (21.0%)	1,160
ASAQ	744 (81.8%)	20 (2.2%)	18 (2.0%)	127 (14.0%)	909
DP	1,242 (91.2%)	13 (1.0%)	22 (1.6%)	85 (6.2%)	1,362

*AL* artemether-lumefantrine , *ASAQ* artesunate-amodiaquine , *DP* dihydroartemisinin-piperaquine

†Percentages are out of total patients treated with that regimen

**Table 2:** Full data estimate of cure at day 28 follow-up using the Kaplan-Meier method and cured proportion

Treatment	Estimate	95% Confidence Interval	SE	SE†
Full data K-M ^a^				
AL	0.960	0.948—0.972	0.0061	0.1559
ASAQ	0.979	0.969—0.989	0.0049	0.2367
DP	0.983	0.977—0.990	0.0035	0.2082
Full data cured proportion ^b^				
AL	0.964	0.951—0.973	0.0056	0.1562
ASAQ	0.980	0.968—0.987	0.0047	0.2357
DP	0.984	0.975—0.989	0.0034	0.2132

*AL* artemether-lumefantrine , *ASAQ* artesunate-amodiaquine , *DP* dihydroartemisinin-piperaquine , *SE* standard error

† Standard error after complementary log-log transformation. The cloglog transformation was applied as the MI estimates were computed on complementary log-log scale for the application of Rubin’s combination rules to be valid.

^a^ For the derivation of the K-M estimates, new infections were considered as censored on the day of recurrence

^b^The estimates of cured proportion (total cured/total number of patients treated) was computed by considering those with new infections as cured. The variance for cured proportion ($\hat{p}$) for a total number of patients (n) was calculated as $\hat{p}\left(1-\hat{p}\right)/n$. The variance was converted to the cloglog scale using the delta method presented in Additional file 1, Section C. The 95% confidence interval was derived using Wilson’s method using binom.confint routine in binom package R.

### Rationale of the simulation design

**Fig. 1:**
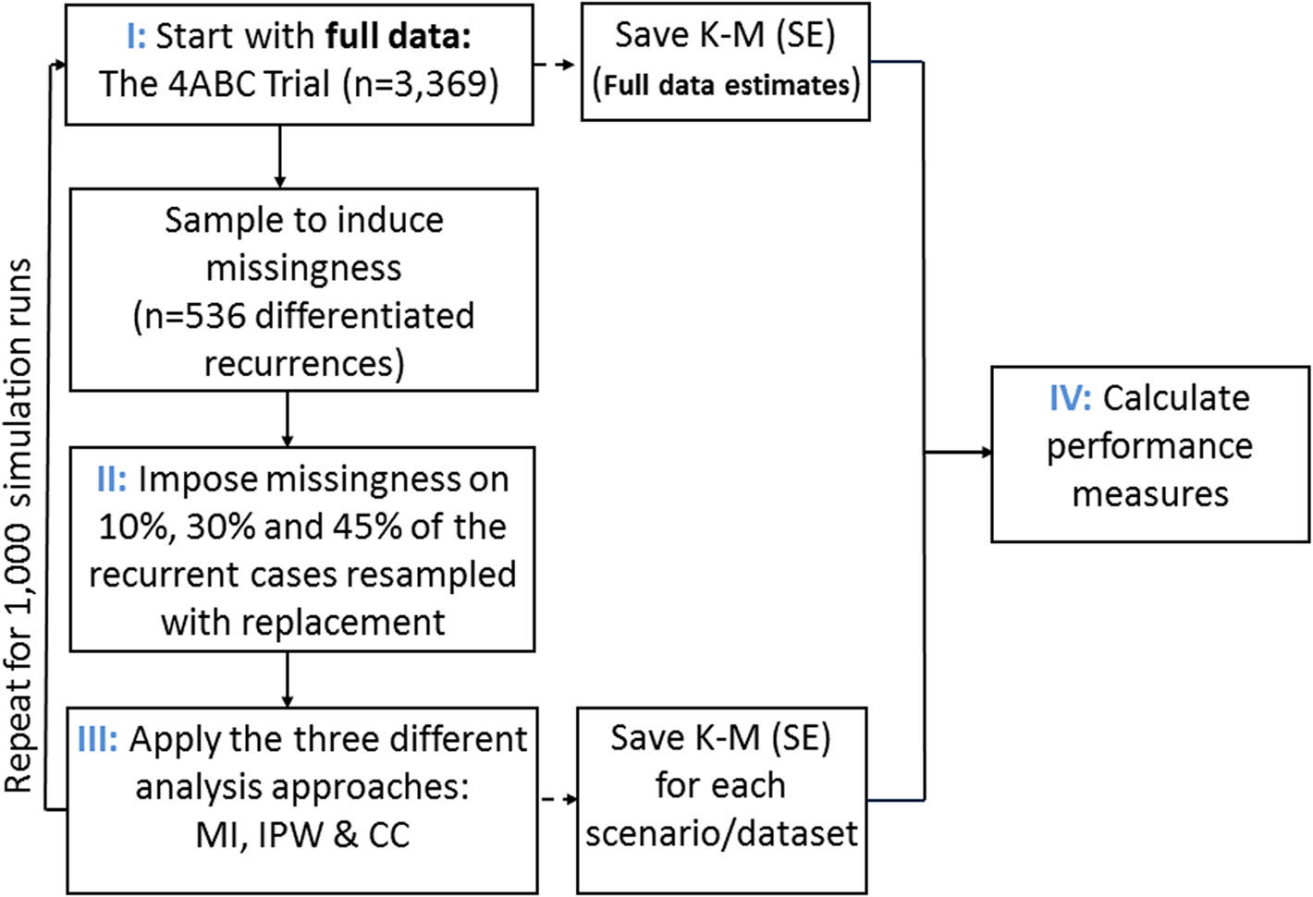
The design of the simulation study. Legend: MI = Multiple Imputation; IPW = Inverse Probability Weighting; K-M = Kaplan-Meier estimate; SE = Standard Error. The truth was defined as the estimates obtained from the full data before missingness was induced

The underlying mechanisms of parasitic recrudescence and new infection represents a complex biological process and simulating data which appropriately reflects this mechanism is difficult. Hence, this simulation study used a real motivational dataset to explore the approaches for handling missing outcome data unique to antimalarial trials. The simulation approach used in this study has been previously described and applied by Brand et al. [20], Rodwell et al. [21], and Rombach et al. [22]. We used the “full data” and simulated the missing data process by repeatedly setting a set proportion of the indeterminant recurrences as missing under two different mechanisms (Fig. 1).

### Mechanisms and models for simulating missingness

The terms missing completely at random (MCAR) and missing at random (MAR) are widely used in the statistical literature to describe the missingness mechanisms. Since missingness of outcomes in antimalarial studies is conditional on a recurrence being observed, we have not used the generic terms of MCAR and MAR when referring to the missing data scenarios simulated in our study. The missing data process was simulated by repeatedly setting a proportion of the recurrent cases to missing under two different mechanisms (**mechanisms 1 and 2**). The following proportions of recurrent cases resampled from the full data were set as missing: 10, 30 and 45% of the full data and for each of these missing fractions, 1000 datasets were simulated. The value of 10% was chosen to mimic the percentage of indeterminate recurrences (among all recurrent infections) observed in the 4ABC trial (a realistic scenario), and 30 and 45% were chosen to represent moderate and extreme scenarios. The overall design of this simulation study is presented in Fig. 1.

For **mechanism 1**, it was posited that missingness was a truly random process among the 536 patients with recurrent infections with 10, 30, and 45% of these patients randomly being reclassified as having indeterminate outcomes [17]. The missingness was induced as follow:

i. For each subject *i* with recurrent parasitaemia, generate a random number (*u*_*i*_) from a uniform distribution [0;1]
  $$ {u}_i\sim U\ \left[0,1\right];i=1,2,3,\dots, 536 $$
ii. Set the desired proportion (*p*) of the smallest *u*_*i*_ as having missing outcome; *p* = 0.10, 0.30, 0.45

For **mechanism 2**, the probability of indeterminate outcome was assumed to depend on transmission intensity and treatment regimen. This assumption was based on regression modelling of the original 4ABC dataset (62 indeterminate recurrences, 81 recrudescences, 455 new infections and 2833 cured) to determine the predictors associated with indeterminate outcomes (see Additional file 1, Section A). Malaria prevalence was estimated from data from the Malaria Atlas Project (MAP) according to latitude, longitude and the year of the study [23]. Transmission settings were categorised as low if MAP estimate were less than or equal to 0.10, moderate if > 0.10 and ≤ 0.40, and high if greater than 0.4. Missingness was induced in a two-step approach as described below:

i) For each subject *i* with recurrent parasitaemia, the probability of their outcome being missing (*π*_*i*_) was estimated using a logistic regression model based on the treatment regimen and the transmission level of the site the subject came from:
  $$ logit\left(\pi \left({\delta}_i=1\right)\right)={\beta}_0+\sum \limits_{k=1}^2{\beta}_{1k}\ast {transmission}_{ik}+\sum \limits_{j=1}^2{\beta}_{2j}\ast {treatment}_{ij} $$
  where *δ*_*i*_ is an indicator variable for missing outcome for individual *i*, and *k* = 1 and 2 for low and moderate transmission respectively (*k* = 0 for high settings as reference category), and *j* = 1 and 2 for antimalarial treatments ASAQ and DP respectively (*j* = 0 for AL as reference arm).
ii) Generate a Bernoulli outcome (*y*_*i*_) for missingness for subject *i* based on the probability of outcome being missing (*π*_*i*_) estimated in step (i) as:

$$ {y}_i\sim Bernoulli\left({\pi}_i\right);i=1,2,3,\dots, 536 $$

Under mechanism 2, two different scenarios were studied representing weak and strong relationship between the covariates and missingness. The coefficients (*β*_1k_; *β*_2*j*_) used for assigning missingness for the **weak scenario (mechanism 2a) and strong scenario (mechanism 2b)** are detailed in Table 3 and the generating model used are given below:

Mechanism 2a (Weak scenario) *logit*(*π*(*δ*_*i*_ = 1)) = *ψ*_0_ + *ψ* where

$$ \psi =\sum \limits_{k=1}^2{\beta}_{1k}\ast {transmission}_{ik}+\sum \limits_{j=1}^2{\beta}_{2j}\ast {treatment}_{ij} $$

and *ψ*_0_ =  − 2.10, − 0.75, − 0.11 for approximately 10, 30, and 45% missingness respectively.

Mechanism 2b (Strong scenario) *logit*(*π*(*δ*_*i*_ = 1)) = *ψ*_0_ + *ψ* where

$$ \psi =\sum \limits_{k=1}^2{\beta}_{1k}\ast {transmission}_{ik}+\sum \limits_{j=1}^2{\beta}_{2j}\ast {treatment}_{ij} $$

and *ψ*_0_ =  − 2.03, − 0.68, − 0.02 for approximately 10, 30, and 45% missingness respectively.

For the strong scenario, the strength of the association between transmission, treatment and missingness was 2-fold higher than that of the weak scenario, to represent a more extreme case. The constant *ψ*_0_ was chosen by iteration to approximately achieve the desired proportions of missing outcomes. Under this generating model, patients treated with ASAQ and DP were progressively less likely to be assigned indeterminate outcomes, reflecting the increasingly longer elimination half-lives with these drug regimens which prevents some of the recurrences to be fully observed by 28 days; and similarly, patients in the moderate and low transmission settings were progressively less likely to have indeterminate outcomes compared to those in high settings as the genotyping method is less likely to fail as clonal competition is lower due to reduced parasitic diversity in low transmission areas.

**Table 3:** Specification of the logistic regression model used to impose missingness

	Log (Odds Ratio)†		Odds Ratio	
	Mechanism 2a	Mechanism 2b	Mechanism 2a	Mechanism 2b
*Treatment*	*β*_2*j*_	*β*_2*j*_	*exp*(*β*_2*j*_)	*exp*(*β*_2*j*_)
AL (reference)	0.00	0.00	1.00	1.00
ASAQ	-0.05	-0.10	0.95	0.90
DP	-0.20	-0.40	0.82	0.67
*Transmission*	*β*_1*k*_	*β*_1*k*_	*exp*(*β*_1*k*_)	*exp*(*β*_1*k*_)
High (reference)	0.00	0.00	1.00	1.00
Low	-0.25	-0.50	0.78	0.61
Moderate	-0.15	-0.30	0.86	0.74

*AL* artemether-lumefantrine , *ASAQ* artesunate-amodiaquine , *DP* dihydroartemisinin-piperaquine

†The intercept of the logistic regression (β_0_) was chosen by iteration to achieve the desired proportion of missingness conditional on recurrence status. This was -2.10, -0.75 and -0.11 for 10%, 30% and 45% respectively under missingness mechanism 2a, and -2.03, -0.68 and -0.02 respectively for 10%, 30% and 45% missingness under mechanism 2b.

### Methods for handling missing data

Three different approaches were used for handling missing data: complete case analysis, multiple imputation and inverse probability weighting. In addition to these three methods, the simulated datasets were also analysed using the analytical approach outlined in equation 2. The estimate of the variance of the analytical solution is presented in Additional file 1 (Section B2). Each of these three methods was applied to the same 1000 independent datasets generated. From each simulated dataset, the target K-M estimates, and associated standard errors were extracted and stored. The construction of the imputation and missingness model is detailed below.

### *Multiple imputation (MI)*

Missing outcomes were imputed using a logistic regression restricted to patients with recurrent parasitaemia (81 recrudescences and 455 new infections) using the MICE algorithm in R. For each observation with simulated missing outcome (*δ*_*i*_ = 1), the missing values were modelled based on the covariate set outlined in the imputation model (Table 4). The imputation model included all the variables in the target analysis, that is, treatment regimen and the observed time to parasite recurrence since the substantive analysis is a survival analysis, plus auxiliary variables identified in the clinical literature [24–27]. In addition, predictors of missingness were also added to the imputation model to reduce the between-imputation variability [8, 28]. Since the study was carried out in multiple centres, study site was also included in the imputation model. Interactions or non-linear relationships between the variables in the imputation model and the missing outcome were not considered. Our approach of using a parametric imputation model (i.e. logistic regression for imputing missing outcome event) and a non-parametric method for carrying out the substantive analysis (i.e. estimating 28 day parasitic recrudescence using the Kaplan Meier function) has been evaluated in other simulation studies with minimal bias observed despite the incompatibility between the imputation and substantive models (Lee et al. (2011), Lee, Dignam and Han (2014)) [29, 30].

The number of imputations (*m* = 50) were selected following the recommendation that *m* should be at least equal to the percentage of missing cases when the fraction of missing information is less than 50% [11, 31]. Since the missingness was restricted to recurrences only (sample size for imputation stage reduced to 284, 145, and 107 for AL, ASAQ and DP respectively), imputation was not performed separately by treatment arms. An overall imputation was carried out by including treatment regimen as a covariate in the imputation model. For each of the analyses, 50 multiply imputed datasets were created and the derived estimates of K-M and associated standard errors were pooled using Rubin’s rules to obtain an overall MI estimate and standard error [14]. Rubin’s combination rules require that the estimated parameter are asymptotically normally distributed [11, 32, 33]. Therefore, the K-M estimates were complementary log-log transformed (cloglog) $\left\{\log \left(-\log \left(\hat{S_{KM}}(t)\right)\right)\right\}$ using Taylor’s series expansion as detailed below (further details in Additional file 1, Section C):

$$ {\displaystyle \begin{array}{c} Var\left\{\mathit{\ln}\left(-\mathit{\ln}\left(\hat{S_{KM}}(t)\right)\right)\right\}\approx {\left\{\frac{1}{\mathit{\ln}\left(\hat{S_{KM}}(t)\right)}\right\}}^2. Var\left(\mathit{\ln}\left(\hat{S_{KM}}(t)\right)\right)\\ {}\Rightarrow Var\left\{\mathit{\ln}\left(-\mathit{\ln}\left(\hat{S_{KM}}(t)\right)\right)\right\}\approx {\left\{\frac{1}{\mathit{\ln}\left(\hat{S_{KM}}(t)\right)}\right\}}^2\frac{1}{\hat{S_{KM}}{(t)}^2}. Var\left(\hat{S_{KM}}(t)\right)\end{array}} $$

**Table 4:** Outline of the imputation and missingness models

Model	Response	Predictors
Imputation Model	$$ Y=\Big\{{\displaystyle \begin{array}{l}1\kern1.25em if\ recrudescence\\ {}0\kern1.25em if\ new\ infection\end{array}}\operatorname{} $$	• age (years) • mg/kg dose of partner drug • transmission intensity ^a^ • treatment regimen • time of recurrence • parasitaemia (log) • study sites ^b^ • parasite density (log) on the day of recurrence
Missingness model	$$ {Y}_{obs}=\Big\{{\displaystyle \begin{array}{l}1\kern1.25em if\ outcome\ is\ observed\\ {}0\kern1em if\ outcome\ is\ indeterminate\end{array}}\operatorname{} $$	• age (years) • mg/kg dose of partner drug • transmission intensity ^a^ • treatment regimen • time of recurrence • recurrence status (yes/no) ^c^

^a^ Transmission settings were categorised as low if Malaria Atlas Project estimate were less than or equal to 0.10, moderate if >0.10 and ≤ 0.40, and high if greater than 0.40

^b^ Study site was not added in the missingness model as it led to convergence issues

^c^ Excluded in the IPW-E approach

### *Inverse Probability Weighting*

A missingness model (selection model) was constructed to estimate the probability of a patient having an observed treatment outcome (cure/recrudescence/new infection) using a logistic regression as specified in Table 4. Patients who were cured (i.e. did not observe recurrence) received a weight of one, while those who had a recurrence status received weights which were the inverse of their estimated missingness probability. As for the imputation model, interactions or non-linear relationships between variables were not considered in the missingness models. For the estimate of the standard errors of the IPW approach to be valid, uncertainties regarding the estimation of the weight should be fully accounted for. Therefore bootstrapping with 200 resamples was undertaken to obtain the standard error as described by Austin et al. 2016 [34]; (see Additional file 1, Section D for a comparison of the standard errors obtained from the naïve approach to the one obtained from bootstrapping method). Efficacy studies for antimalarials are unique in that indeterminate outcomes can arise only if a patient experiences parasitic recurrence- and thus recurrence is a perfect predictor. Two different strategies for handling this perfect predictor were considered; by including (IPW) and excluding it (IPW-E) in the missingness model (Table 4).

### Performance measures for evaluating different methods

Let *θ* be the true value of the “fixed” estimand of interest derived from the full dataset and $\hat{\theta_s}$ is the estimate of *θ* generated from the *s*^th^ simulation. The estimand of primary interest was the Kaplan-Meier estimate of 28 day parasitic recrudescence, $\hat{S_{KM}}(t)$, derived from the full data (shown in Table 2) (which considered new infection as censored). In addition, a second estimand of interest was cured proportion (which considered new infection as success) (Table 2). The performance measures of the derived estimator were assessed in terms of bias, efficiency and coverage compared to the “true estimands” as described in Table 6 of Morris et al. [35].

Bias was defined as the difference between the average of the estimates ($\hat{\theta_s}\Big)$ obtained from the 1000 datasets with simulated missingness and the ‘fixed’ full data estimate (*θ*). The bias was expressed as relative percentage bias, which is bias relative to the full data estimate $:\left(\frac{bias}{\theta}\right)\times 100\%$. Model based standard error (ModSE) was calculated as the square root of the average variance across 1000 datasets and the empirical standard error (EmpSE) was calculated as the square root of the variance of the estimated K-M across 1000 datasets. Root mean squared error (RMSE), which combines the bias and variance of the estimate, was reported as a measure of overall accuracy. The expression for bias, ModSE and EmpSE are given below:

$$ \mathrm{Bias}=\frac{1}{n_{sim}}\sum \limits_{s=1}^{n_{sim}}\left(\hat{\theta_s}-\theta \right)\kern2.3em ;{n}_{sim}=1,000 $$

$$ \mathrm{ModSE}=\sqrt{\frac{1}{n_{sim}}\sum \limits_{s=1}^{n_{sim}}\hat{Var\Big(\hat{\theta_s\Big)}}}\kern0.5em ;{n}_{sim}=1,000 $$

$$ \mathrm{EmpSE}=\sqrt{\frac{1}{\left({n}_{sim}-1\right)}\sum \limits_{s=1}^{n_{sim}}{\left(\hat{\theta_s}-\overline{\theta}\right)}^2};{n}_{sim}=1,000 $$

$$ \mathrm{RMSE}=\sqrt{\frac{1}{n_{sim}}\sum \limits_{s=1}^{n_{sim}}{\left(\hat{\theta_s}-\theta \right)}^2}\kern0.5em ;{n}_{sim}=1,000 $$

The coverage probability was estimated as the proportion of the 1000 datasets where the estimated 95% confidence interval (CI) included the point estimate of the K-M derived from the full dataset (‘fixed’ full data estimate) before inducing missingness. For a 95% confidence interval, the theoretical coverage is expected to fall between 93.6 to 96.4% with 1000 simulated datasets. A drop in coverage below 90% is regarded as problematic [28]. Finally, Monte Carlo standard error (MCSE), which represents the noise attributable to the finite number of simulations used was reported for each of the performance measures reported [36].

## Software

Multiple imputation was carried out using mice library and Kaplan-Meier estimates were generated using survival library in R statistical software [37].

## Results

There were a total of 598 (17.4%, 598/3431) recurrences of which 81 were recrudescences, 455 new infections and 62 indeterminate outcomes after performing genotyping (Table 1). While the percentage of indeterminate outcomes out of the total sample size is low (1.8%, 62/3431), this represents 10.4% (62/598) of the total recurrences. For the purpose of the analysis, 62 indeterminate outcomes were excluded and the remaining 3369 study participants with molecularly defined parasitic recurrence were used to represent a complete dataset (full data). Of these, 49% (1645) of patients were from areas of high transmission, 31% (*n* = 1033) from moderate and 21% (*n* = 691) from the areas of low transmission settings from a total of 10 different study sites. The median baseline parasitaemia was 28,855/μL and was similar between the treatment regimens. On the recurrence day, the median parasitaemia was 6080/μL for recrudescences and 4600/μL for new infections.

### Performance measures

**Fig. 2:**
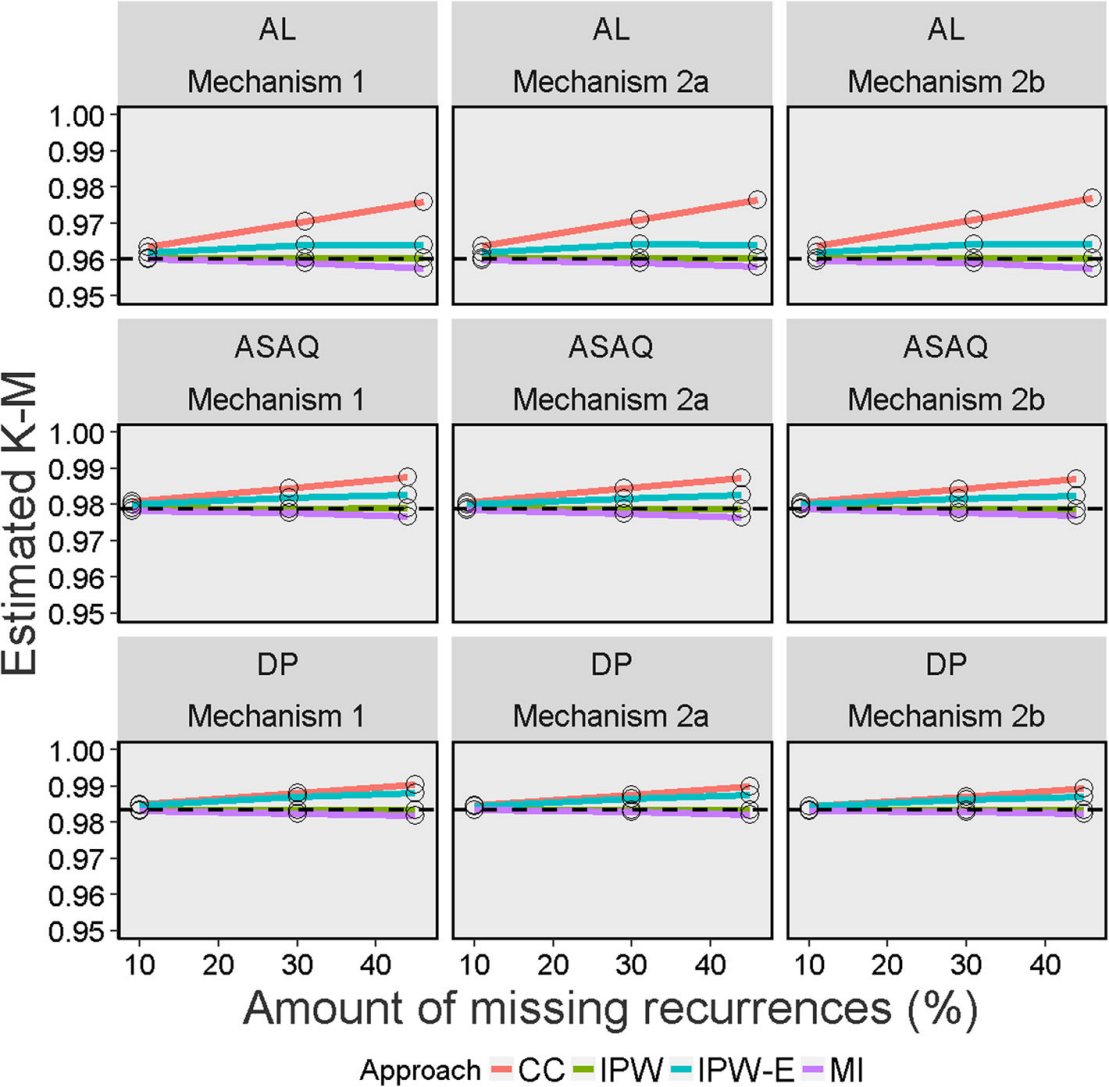
Average estimate of Kaplan-Meier survival probability on day 28 across 1000 simulated datasets for different missing data methods, missing data mechanisms and percentages of indeterminate recurrences Legend: CC = Complete Case; IPW = Inverse Probability Weighting; IPW-E = Inverse Probability Weighting with recurrence status excluded; MI = Multiple Imputation; AL = artemether-lumefantrine; ASAQ = artesunate-amodiaquine; DP = dihydroartemisinin-piperaquine. The dotted line represents the estimates derived from the full data estimate before missingness was induced

**Fig. 3:**
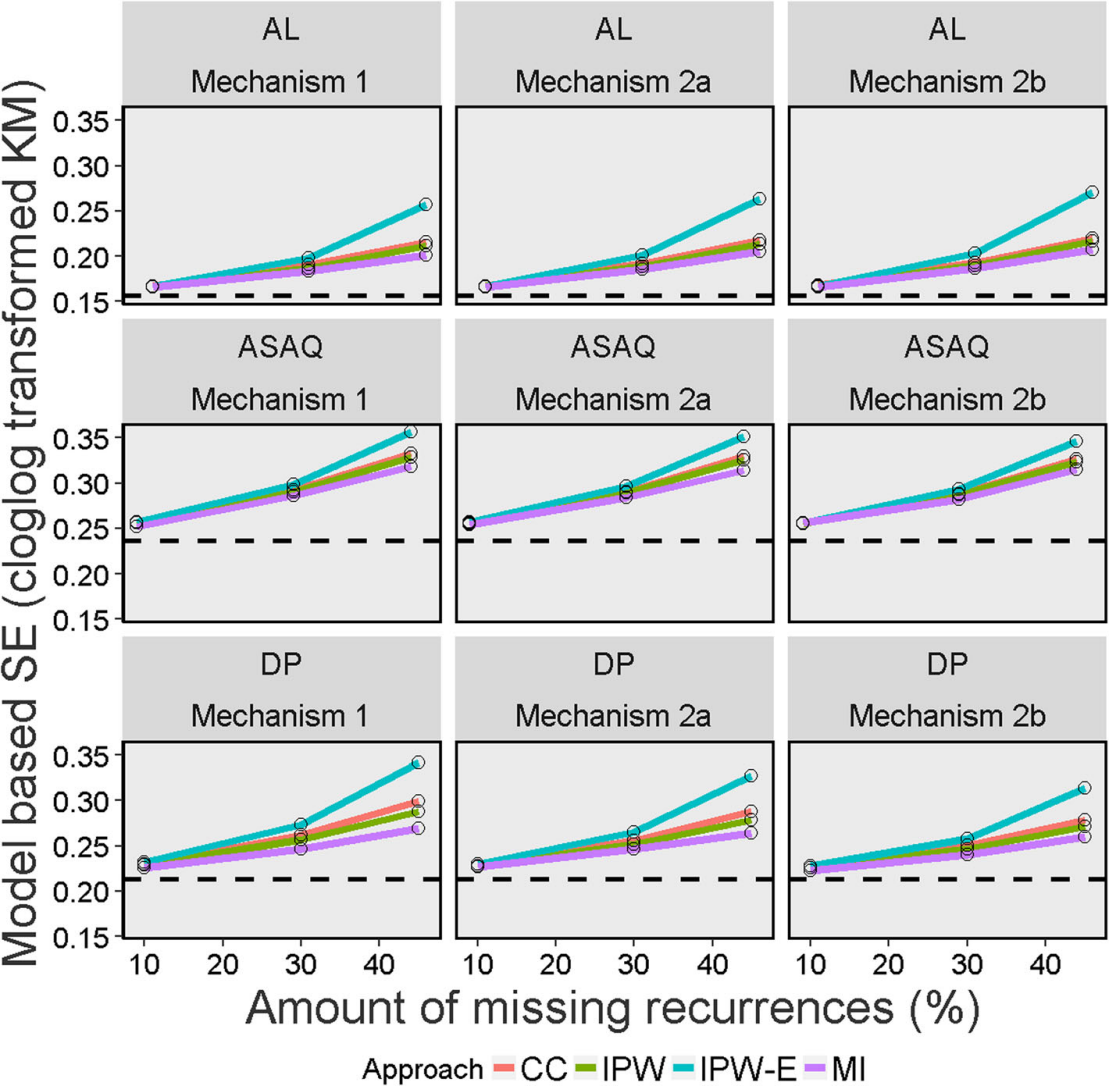
Model based standard errors of Kaplan-Meier estimates on day 28 across 1000 simulated datasets for different missing data methods, missing data mechanisms and percentages of indeterminate recurrences (on complementary log-log scale) Legend: CC = Complete Case; IPW = Inverse Probability Weighting; IPW-E = Inverse Probability Weighting with recurrence status excluded; MI = Multiple Imputation; AL = artemether-lumefantrine; ASAQ = artesunate-amodiaquine; DP = dihydroartemisinin-piperaquine. The dotted line represents the full data estimate before missingness was induced (estimate of true value)

**Fig. 4:**
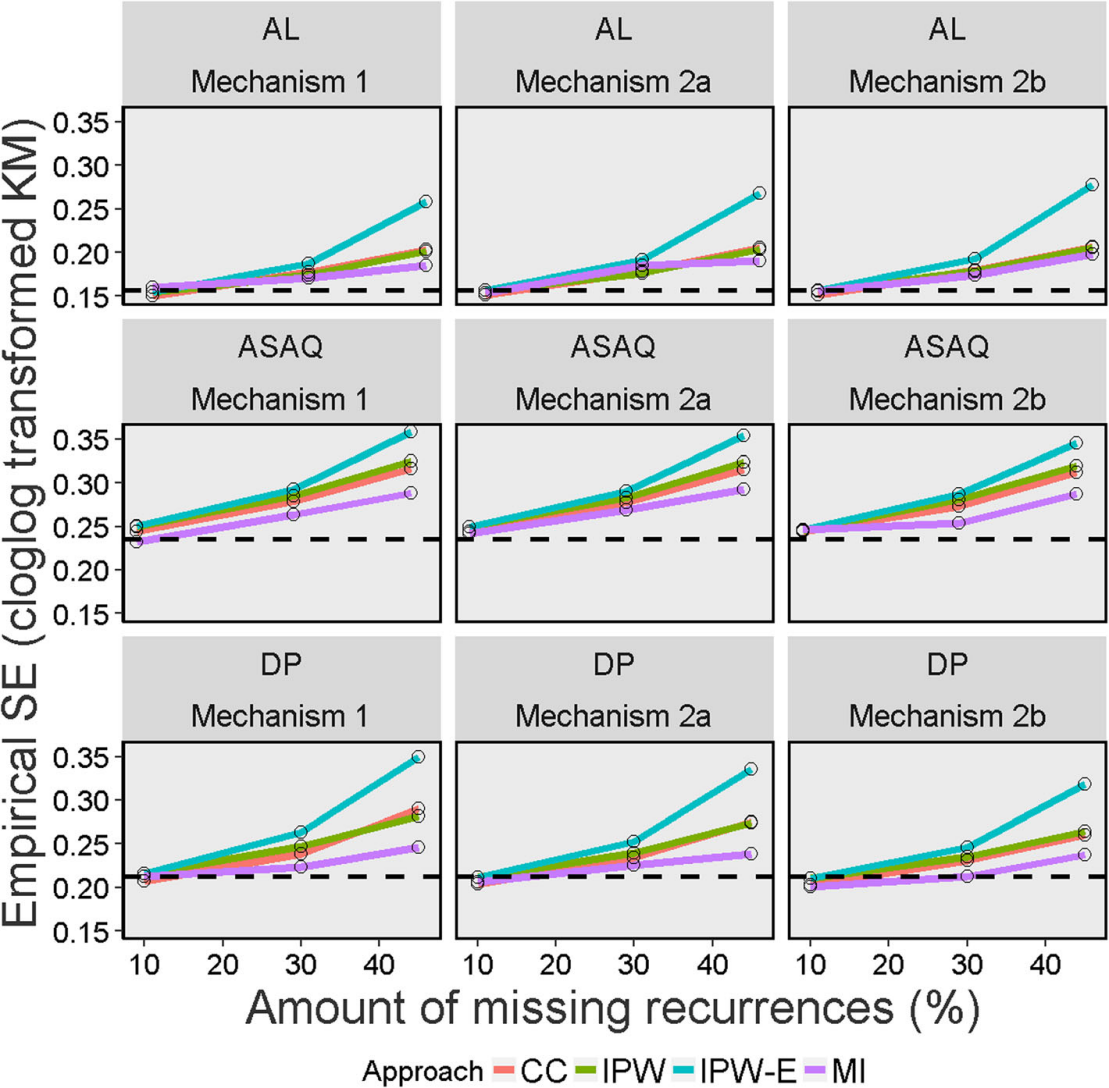
Empirical standard errors of Kaplan-Meier estimates on day 28 across 1000 simulated datasets for different missing data methods, missing data mechanisms and percentages of indeterminate recurrences (on complementary log-log scale) Legend: CC = Complete Case; IPW = Inverse Probability Weighting; IPW-E = Inverse Probability Weighting with recurrence status excluded; MI = Multiple Imputation; AL = artemether-lumefantrine; ASAQ = artesunate-amodiaquine; DP = dihydroartemisinin-piperaquine. The dotted line represents the full data estimate before missingness was induced (estimate of true value)

**Fig. 5:**
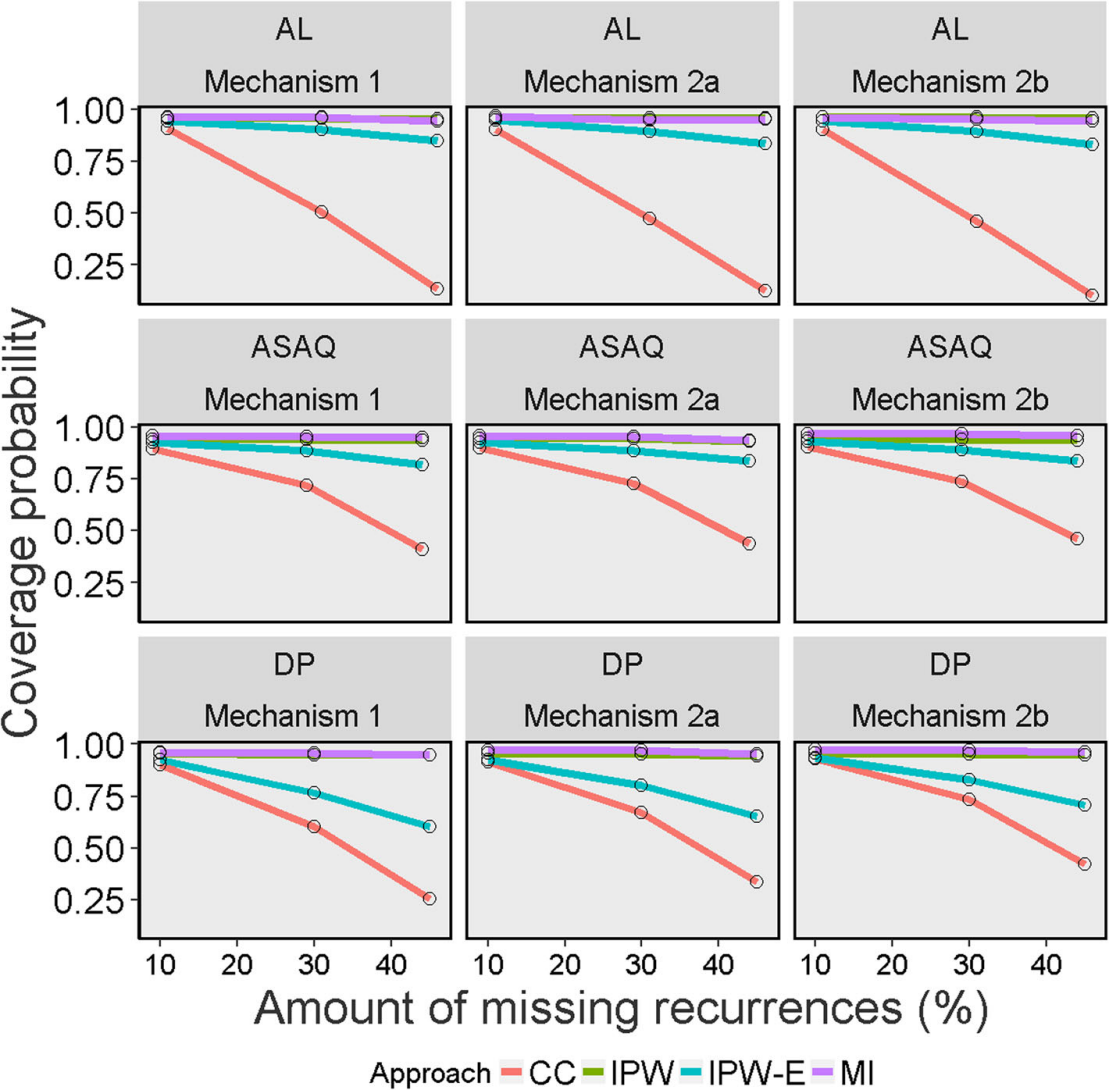
Coverage probability of Kaplan-Meier estimates on day 28 across 1000 simulated datasets for different missing data methods, missing data mechanisms and percentages of indeterminate recurrences Legend: CC = Complete Case; IPW = Inverse Probability Weighting; IPW-E = Inverse Probability Weighting with recurrence status excluded; MI = Multiple Imputation; AL = artemether-lumefantrine; ASAQ = artesunate-amodiaquine; DP = dihydroartemisinin-piperaquine

**Fig. 6:**
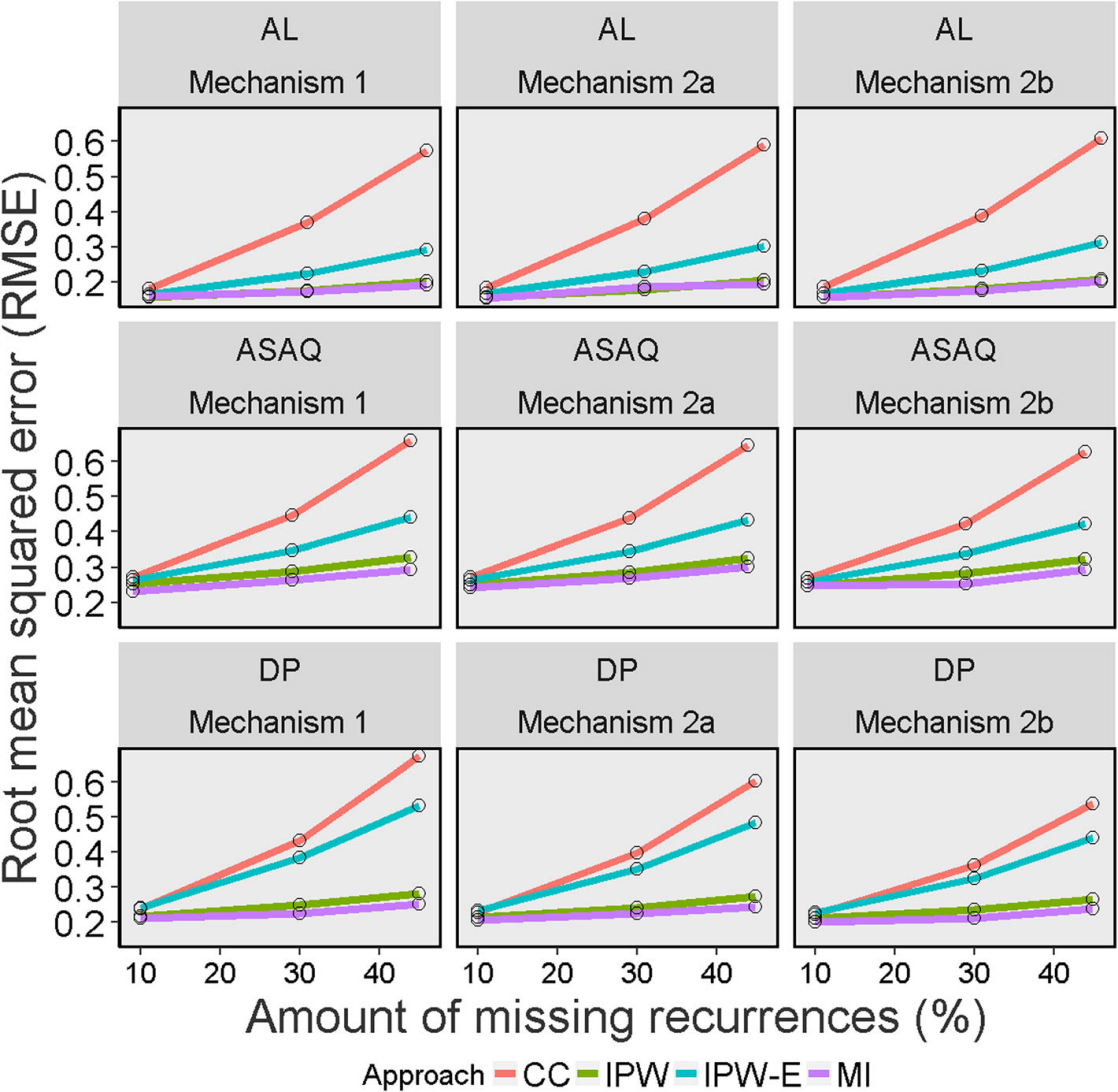
Overall accuracy of Kaplan-Meier estimates on day 28 across 1000 simulated datasets for different missing data methods, missing data mechanisms and percentages of indeterminate recurrences Legend: CC = Complete Case; IPW = Inverse Probability Weighting; IPW-E = Inverse Probability Weighting with recurrence status excluded; MI = Multiple Imputation; AL = artemether-lumefantrine; ASAQ = artesunate-amodiaquine; DP = dihydroartemisinin-piperaquine

The result of the different performance measures for the different methods used for handling missing data are presented in Tables 5 and 6, Figs. 2, 3, 4, 5 and 6, and in Section E of Additional file 1. Since the “true values” from the full data set before inducing missingness were slightly different for the K-M estimate (which considered new infection as censored) and cured proportion (which considered new infection as success) (Table 2), the performance measures are discussed separately for these two estimands. As the primary aim of this simulation was to evaluate the performance measures of different missing data approaches in deriving K-M estimates, much of the results and discussion is focussed on this estimand.

**Table 5:** Performance measures of various methods for handling 45% missingness in recurrences for individuals treated with artemether-lumefantrine

Full data Kaplan-Meier estimate of day 28 cure (SE) = 0.960 (0.1559)				
	Complete case analysis	MI	IPW	IPW-E
Mechanism 1				
Bias	0.0159 (0.0165)	-0.0026 (0.0079)	0.0002 (0.0075)	0.0039 (0.0097)
Relative bias	1.65 %	-0.27 %	0.02 %	0.41 %
Model based SE	0.2152 (0.0007)	0.2001 (0.0008)	0.2097 (0.0009)	0.2536 (0.0014)
Empirical SE	0.2032 (0.0045)	0.1854 (0.0041)	0.2015 (0.0045)	0.2582 (0.0058)
Coverage	13.4 % (1.1)	94.4 % (0.7)	95.4 % (0.7)	85.1 % (1.1)
RMSE ^a^	0.5732 (0.0079)	0.1914 (0.0014)	0.2028 (0.0023)	0.2918 (0.0044)
Mechanism 2a (Weak scenario)				
Bias	0.0163 (0.0173)	-0.0022 (0.0084)	0.0001 (0.0077)	0.0039 (0.0104)
Relative bias	1.70 %	-0.23 %	0.01 %	0.41 %
Model based SE	0.2176 (0.0008)	0.2041 (0.0008)	0.2123 (0.0009)	0.2594 (0.0017)
Empirical SE	0.2053 (0.0046)	0.1901 (0.0044)	0.2028 (0.0046)	0.268 (0.0062)
Coverage	12.3 % (1.0)	95.5 % (0.7)	95.7 % (0.6)	83.7 % (1.2)
RMSE ^a^	0.5907 (0.0085)	0.1938 (0.0018)	0.2039 (0.0024)	0.3019 (0.0051)
Mechanism 2b (Strong scenario)				
Bias	0.0167 (0.0169)	-0.0026 (0.008)	0.0001 (0.0076)	0.004 (0.0102)
Relative bias	1.74 %	-0.27 %	0.01 %	0.41 %
Model based SE	0.2200 (0.0008)	0.2060 (0.0008)	0.2151 (0.0009)	0.2659 (0.0016)
Empirical SE	0.2060 (0.0046)	0.1978 (0.0043)	0.2051 (0.0045)	0.2772 (0.006)
Coverage	10.1 % (1.0)	94.5 % (0.7)	95.7 % (0.6)	83.1 % (1.2)
RMSE ^a^	0.6085 (0.0082)	0.2031 (0.0016)	0.2063 (0.0023)	0.3119 (0.0046)

*MI* Multiple Imputation, *IPW* Inverse Probability Weighting, *IPW-E* Inverse Probability Weighting with recurrence status excluded; SE= Standard Error; RMSE= Root Mean Squared Error; K-M = Kaplan-Meier estimates

^a^=Monte Carlo error for the RMSE presented on mean squared error scale

Monte Carlo Standard Errors shown in parentheses

**Table 6:** Performance measures of complete case and maximum likelihood estimator for handling 45% missingness in recurrences for individuals treated with artemether-lumefantrine in estimating day 28 cured proportion

Full data estimate of day 28 cure proportion (SE) = 0.9637 (0.1561) ^a^		
	Complete Case analysis	Maximum Likelihood Estimator
Mechanism 1		
Bias	1.3724 (0.0145)	0.0000 (0.0075)
Relative Bias	1.42%	-0.00%
Model based SE	0.2153 (0.0007)	0.2100 (0.0008)
Empirical SE	0.2177 (0.005)	0.2159 (0.0048)
Coverage	21.4% (1.3%)	93.1% (0.8%)
RMSE ^b^	0.5503 (0.0077)	0.2169 (0.0022)
Mechanism 2a (Weak scenario)		
Bias	1.4093 (0.0149)	-0.0004 (0.0077)
Relative Bias	1.46%	-0.04%
Model based SE	0.2179(0.0008)	0.2123 (0.0008)
Empirical SE	0.2227 (0.0049)	0.2185 (0.0049)
Coverage	19.2% (1.2%)	93.4% (0.8%)
RMSE ^b^	0.5686 (0.0082)	0.2186 (0.0023)
Mechanism 2b (Strong scenario)		
Bias	1.4437 (0.0152)	-0.0009 (0.0079)
Relative Bias	1.50%	-0.09%
Model based SE	0.2202 (0.0008)	0.2143 (0.0008)
Empirical SE	0.2248 (0.0050)	0.2204 (0.0049)
Coverage	17.2% (1.2%)	93.8% (0.8%)
RMSE ^b^	0.5844 (0.0084)	0.2203 (0.0023)

*RMSE* Root Mean Squared Error, *AL* artemether-lumefantrine, *AS-AQ* artesunate-amodiaquine, *DP* dihydroartemisinin-piperaquine

^a^ The “true” estimates of cured proportion (total cured/total number of patients treated) before missingness was induced. Those with new infections were counted as cured. The variance for cured proportion (p) for a total number of patients (n) was calculated as *p*(1 − *p*)/*n*. The variance was converted to the cloglog scale using the equation presented in Additional file 1, Section C.

^b^ Monte Carlo error for the RMSE presented on mean squared error scale. Monte Carlo Standard Errors shown in parentheses

### Bias

In all of the scenarios studied, the CC analysis resulted in an upwards biased estimate of day 28 K-M estimate of probability of cure, which was incremental with increasing missingness, irrespective of the treatment regimen and missingness mechanism studied. For example, in the AL arm, the bias was 0.34, 1.04 and 1.59% when respectively 10, 30 and 45% of the recurrences were set to missing under mechanism 1. The IPW approach, which included recurrence status as a predictor (IPW) in the missingness model provided the most consistent estimate of all approaches (Table 5). IPWs calculated from a missingness model that excluded recurrence status (IPW-E) produced larger biases compared to standard IPW and MI approaches. In general, MI estimates were slightly negatively biased whereas the IPW estimates exhibited positive bias. The MI and IPW approaches led to smaller biases under mechanisms 2a and 2b compared to mechanism 1, although this magnitude was negligible. Similarly, there was no clear trend in the direction of bias for mechanisms 2a and 2b. The MC error, which is the noise from the finite number of simulations, didn’t exceed 0.008 for MI and IPW (Table 5).

The analytical solution outlined in equation 2 was a consistent estimator of the cured proportion whereas CC approach was upwards biased in all scenarios studied (Table 6 and Additional file 1 Tables 7, 8, 9).

### Model based and empirical standard errors

The variance of the K-M estimates increased as the proportion of missing outcomes increased for all the approaches used for handling missing data. When 10% of the recurrences were missing, estimates of model based SE were similar across the methods, with differences observed only at the third decimal place. When missingness was ≥30%, there was a clear trend in efficiency. IPW-Exclude had the largest SE followed by the CC analysis. MI and standard IPW had smaller standard errors compared to other approaches with MI performing the best across all scenarios studied. It was also found that the IPW implementation which didn’t fully account for uncertainty associated with estimating weights (naïve estimator) resulted in SEs which were (falsely) smaller than the MI estimates. The comparison of SEs for IPW using the naïve approach and bootstrap method are presented in Additional file 1 (See Section D). The average gain in precision (model based SE) with IPW compared to the CC analysis over all missingness mechanism were 1.9, 4.7, and 6.9% for 10, 30 and 45% of missingness respectively. With MI, these were 2.9, 9.0, 16.9% for 10, 30 and 45% missingness respectively. Similar results were observed with empirical SEs.

Like with the K-M estimates, the model based SEs and empirical SEs were progressively larger with increasing proportion of missingness for the analytical solution for estimating cured proportion (Table 6). At 10% missingness, the EmpSE and ModSE were similar across the two methods. At 30% or larger missing proportion, there was a small gain in precision with the analytical solution compared to the CC method (Table 6 and Additional file 1 Tables 7, 8, 9).

### Coverage probability of the true value

Figure 5 shows the coverage probability for different missing data methods in derivation of K-M estimates for different missingness proportion. The CC approach suffered from poor coverage in all the scenarios under consideration and this deteriorated rapidly with increasing proportion of missingness. The IPW and MI implementation resulted in coverage probability close to the nominal 95% level irrespective of the missingness scenarios studied. The IPW-Exclude approach was also associated with coverage that was lower than the nominal level across the entire simulation scenarios studied. The MC error for the coverage ranged between 0.5 to 1.6%. For the analytical solution (equation 2), the coverage ranged from 93.1–93.8% across different scenarios whereas the CC estimator for cured proportion suffered from substantial under-coverage (Table 6 and Additional file 1 Tables 7, 8, 9).

### Root mean squared error (RMSE)

The CC approach had the least overall accuracy of all the missing data methods followed by IPW-Exclude across all missingness mechanisms. For all the missing data methods, the overall accuracy decreased with increasing proportion of missingness. MI and IPW approach both provided similar estimates of accuracy. With MI and IPW approach, the accuracy was higher under missingness mechanism 2 compared to the mechanism 1. The overall accuracy of the estimator is presented in Fig. 6. Similarly, the CC approach for estimating cured proportion had the largest RMSE whereas the analytical solution (equation 2) had superior overall accuracy for estimating the cured proportion (Table 6 and Additional file 1 Tables 7, 8, 9).

## Discussion

Missing data in clinical trials can pose analytical challenges, including undermining the validity and interpretation of the results. In antimalarial studies, indeterminate recurrences resulting from genotyping failure are frequently encountered, especially in the areas of high transmission intensity, where multiple infections are common. Principled approaches for handling missing data have proliferated the medical and statistical literature in recent years [9, 10, 38], yet the most common approach used by malaria researchers and recommended by the WHO for handling indeterminate cases is to simply exclude these from the analysis. In this article, the performance of MI and IPW were evaluated for handling indeterminate outcomes in the context of estimation of antimalarial efficacy using one of the largest antimalarial studies (the 4ABC study) [19]. The use of a real dataset to represent the complete (full) data avoided arbitrary choices usually made in simulating covariates and survival data, and provided a rich dataset from multiple endemic settings, with auxiliary covariates for implementation of IPW and MI approaches, thus making the generalisability of results for antimalarial trials more plausible.

Two different missingness mechanisms were investigated and differences in estimates compared for scenarios in which 10, 30 and 45% of the known recurrences were reclassified as missing. In all these scenarios, the current recommendation of excluding indeterminate cases resulted in an upwards biased estimate of day 28 probability of cure (K-M method) by up to a maximum of 1.7% (see Additional file 1, Section E), the magnitude of which was correlated with the proportion of recurrent outcomes classified as indeterminate. Similar findings were observed in Machekano et al. (2008) who reported an absolute overestimation in efficacy of 3.2% by CC approach compared to IPW and MI methods for the antimalarial regimen of chloroquine (CQ) + sulphadoxine-pyrimethamine (SP) and by up to 1.7% for the regimen amodiaquine (AQ) + SP when the observed proportion of missing recurrences were 33% in the CQ + SP arm and 17% for AQ + SP arm [16]. Like for the estimation of the K-M of probability of cure, the CC analysis was associated with overestimation of proportion cured at day 28. The analytical solution outlined in equation 2 provided the most consistent estimate of the proportion cured compared to the CC estimator.

For the derivation of K-M estimate of day 28 probability of cure, MI and IPW approaches were generally consistent under all missingness scenarios and resulted in an increased precision. The IPW approach provided consistently the least biased estimate of K-M probability of cure of all the approaches for all proportions of missingness; however, it came at a price of marginally inflated standard errors compared to the MI approaches which also corroborate well with the observations of Machekano and colleagues [16]. However, the current study had two important differences. First, the Kaplan-Meier method, which is currently the preferred approach for estimating drug efficacy, was used (as opposed to the proportion cured reported in Machekano and colleagues). Second, when constructing the missingness model for the IPW implementation, recurrence status was included as a predictor in this analysis. In antimalarial studies, a missing outcome is only possible once a patient experiences recurrent parasitaemia, thus leading to a scenario where recurrence status is a predictor of missing outcome. It was found that the IPW approach where missingness models excluded the predictor recurrence was associated with an increased bias and inflated standard error. This suggests that recurrence should always be included in the missingness model to obtain valid inferences for the IPW estimate.

Like for the IPW, the validity of the inferences derived from MI relies on the correct implementation of the imputation model, hence this approach should include the correct functional form and specify any interactions. Failure to do so could lead to invalid inferences being drawn, especially when the fraction of missing information is large [11, 31, 39]. In practice, all imputation models are likely to be mis-specified to some extent. Arguably specifying the missingness model correctly is an easier task compared to specifying a correct imputation model [9, 40], thus making the IPW approach a feasible alternative for handling indeterminate outcomes in estimation of efficacy in antimalarial studies. However, it is important to account for the uncertainty associated with estimation of weights in IPW as the naïve estimate of the standard error ignores this uncertainty, leading to the IPW approach paradoxically appearing far more efficient than MI (See Additional file 1, Section D) [34]. In addition to being biased and inefficient, the CC estimates also suffered from poor coverage for the estimation of K-M probability compared to MI and IPW methods and for estimation of cured proportion compared to the analytical solution (equation 2). When the missingness was greater than 30%, the coverage for CC approach deteriorated rapidly and fell below 90% for all the missingness mechanisms (regardless of choice of the estimand) whereas for MI, IPW and the analytical approach, the coverage remained near the nominal 95% level.

The current WHO guidelines require that a new regimen should demonstrate at least 95% efficacy to be included in the antimalarial treatment policy, and further investigations are warranted when treatment failure exceeds 10% to examine the possibility of drug resistance [2]. The results of this study, taken together with the findings of Machekano et al. [16] highlights that CC approach provides an optimistic view of the treatment efficacy and this can have potentially deleterious consequences when the estimate is at the cusp of these WHO thresholds (in a study where a large proportion of outcomes are indeterminate). From a public health perspective, the false sense of confidence generated from these studies regarding the current status of antimalarial regimens can have important ramifications for the evolution of antimalarial drug resistance. The prolonged usage of a less optimal regimen provides a constant drug selection pressure to the parasites; a scenario highly conducive for emergence of de novo drug resistance. Given the paucity of alternative regimens currently available and the spread of artemisinin resistance across South East Asia [41], it is important that researchers and policy makers alike are aware of the pitfalls associated with the CC estimate of efficacy when drawing conclusions from routine surveillance studies. The analytical solution outlined in equation 2 provided the most consistent estimate of the failure and could be a useful alternative in scenarios where there is minimal or no censoring. However, when there is censoring (due to lost-to-follow up or when new infection is considered as censored), the K-M approach through the use of principled approaches of MI and IPW would be the most appropriate method for estimation of the day 28 proportion of recrudescences.

This simulation study has a number of limitations. First, it was assumed that the genotyping outcome reflects the true treatment outcomes. The genotyping procedure is prone to misclassification error, particularly in areas of intense transmission where polyclonal infections present formidable challenge [42–45]. A thorough consideration of genotyping adjusted efficacy should incorporate the population allele diversity, which is often unmeasured or not presented; however the potential confounding from this remains beyond the scope of the current analysis. Second, IPW and MI are not the only available approaches for handling missing data. Likelihood based approaches, which use expectation-maximisation (EM) algorithms are alternative approaches, but at present are not implemented in standard software [46]. The pseudo-value method is increasingly being used and its utility in the context of antimalarial research is yet to be evaluated [47–50]. Third, this simulation study has evaluated the performance of MI and IPW approaches in derivation of K-M estimates and the application of these principled methods for other statistical approaches for estimating efficacy (e.g: competing risk survival analysis approach) was not considered [51–53]. Finally, this study doesn’t represent every missing-data problem which can be encountered in practice and a single method cannot be universally recommended but rather the choice of the method should be guided by the research question and the context of the study.

In the presence of missing data, no statistical methods, simple or sophisticated, can supersede the result, which could have been derived had the data been fully observed. Thus best possible efforts should be made to minimise the missingness through careful design, study management, and adherence to standardised protocols [54–57]. Diligence in sample collection in the field, use of better genotyping method (e.g. capillary based) including appropriate quality control measures through a regular proficiency testing program should be deployed [58]. Missing data should be anticipated in advance and researchers should strive to collect data on variables which might be related to variables expected to exhibit missing data such as background allelic frequency. When using MI and IPW, researchers should clearly report the details of modelling approaches including the construction of imputation and missingness models [8, 59].

The definition of recrudescence and new infection depends on the how different sized bands are binned and classified as being the same or different alleles. For example, Cattamanchi et al. (2003) [60] considered the alleles to be the same if the molecular weights were within 10 base-pair length for merozoite surface protein (*msp)*-2 genes whereas Rouse et al. (2008) reported that an identical *msp*-2 allele could be different by up to 18 base pairs [61]. The definition adopted for defining recrudescence or a new infection is critical and researchers should always endeavour to publish the fragment length of the alleles in the pre- and post-treatment samples as done by Plucinski et al. (2017) (see Additional file 1: Table 1 of [62]).

## Conclusions

The widely used approach of excluding indeterminate outcomes results in underestimation of antimalarial failure. In the example studied, the incorporation of missing data through correctly implemented IPW (including recurrence status as the predictor and using bootstrapping to estimate the standard error) and MI approaches and the analytical solution outlined in equation 2 greatly reduced bias. The IPW and MI approaches were associated with the smallest standard errors and provided superior coverage probability of the derived estimates of day 28 recrudescence. IPW and MI approaches are easily implementable in standard statistical software and should be considered for handling indeterminate outcomes in the derivation of antimalarial failure.

## Supplementary information

**Additional file 1: Section A**: Estimating treatment efficacy for antimalarial drugs; **Section B1**: Quantifying bias in complete case estimator; **Section B2**: Variance of the maximum likelihood estimator; **Section C**: Application of Rubin’s combination rules for pooling multiply imputed Kaplan-Meier estimates; **Section D**: Comparison of naïve and bootstrapped standard error for inverse probability weighting approach; **Section E**: Additional results on performance measures for the simulation study. **Table** **S1.** Antimalarial treatment outcomes for the 4ABC Trial [19] **Table** **S2.** Full data estimate of cure at day 28 follow-up using the Kaplan-Meier method **Table** **S3.** Specification of the logistic regression model used to impose missingness **Table** **S4.** Outline of the imputation and missingness models **Table** **S5.** Performance measures of various methods for handling 45% missingness in recurrences for individuals treated with artemether-lumefantrine **Table** **S6.** Performance measures of complete case and maximum likelihood estimator for handling 45% missingness in recurrences for individuals treated with artemether-lumefantrine in estimating day 28 cured proportion **Figure S1.** Therapeutic responses post antimalarial treatment in *P. falciparum* malaria. Adapted from White NJ: The assessment of antimalarial drug efficacy. *Trends Parasitol* 2002, 18:458–464.^9^

## Data Availability

Data generated and analysed for this study is available from the corresponding author on reasonable request

## Abbreviations

AL: Artemether-Lumefantrine
ASAQ: Artesunate-Amodiaquine
CC: Complete Case Analysis
DP: Dihydroartemisinin-Piperaquine
EmpSE: Empirical Standard Error
IPW: Inverse Probability Weighting
IPW-E: Inverse Probability Weighting with recurrence status excluded
${\hat{S}}_{KM}(t)$: Kaplan-Meier estimates of drug efficacy at time t
K-M: Kaplan-Meier
MI: Multiple Imputation
ModSE: Model based Standard Error
RMSE: Root Mean Squared Error
