# Supplementary material for: Dealing with indeterminate outcomes in antimalarial drug efficacy trials: a comparison between complete case analysis, multiple imputation and inverse probability weighting
Source: BMC Med Res Methodol. 2019 Nov 27;19:215. doi: 10.1186/s12874-019-0856-z (PMC6882216; doi:10.1186/s12874-019-0856-z)
Supplement: Supplementary file 1 — Additional file 1: Section A: Estimating treatment efficacy for antimalarial drugs; Section B1: Quantifying bias in complete case estimator; Section B2: Variance of the maximum likelihood estimator; Section C: Application of Rubin’s combination rules for pooling multiply imputed Kaplan-Meier estimates; Section D: Comparison of naïve and bootstrapped standard error for inverse probability weighting approach; Section E: Additional results on performance measures for the simulation study. Table S1. Antimalarial treatment outcomes for the 4ABC Trial [19] Table S2. Full data estimate of cure at day 28 follow-up using the Kaplan-Meier method Table S3. Specification of the logistic regression model used to impose missingness Table S4. Outline of the imputation and missingness models Table S5. Performance measures of various methods for handling 45% missingness in recurrences for individuals treated with artemether-lumefantrine Table S6. Performance measures of complete case and maximum likelihood estimator for handling 45% missingness in recurrences for individuals treated with artemether-lumefantrine in estimating day 28 cured proportion Figure S1. Therapeutic responses post antimalarial treatment in P. falciparum malaria. Adapted from White NJ: The assessment of antimalarial drug efficacy. Trends Parasitol 2002, 18:458–464.9 [file 12874_2019_856_MOESM1_ESM.docx]

**Additional file 1:**

**Dealing with indeterminate outcomes in antimalarial drug efficacy trials: A comparison between complete case analysis, multiple imputation and inverse probability weighting**

Prabin Dahal^1,2^*, Kasia Stepniewska^1,2^, Philippe J Guerin^1,2^, Umberto D’Alessandro^3,4^, Ric N Price^1,2,5^, Julie A Simpson^6^*

^1^WorldWide Antimalarial Resistance Network (WWARN), Oxford, UK

^2^Centre for Tropical Medicine and Global Health, Nuffield Department of Clinical Medicine, University of Oxford, Oxford, UK

^3^Medical Research Council Unit, Fajara, The Gambia

^4^Unit of Malariology, Institute of Tropical Medicine, Antwerp, Belgium

^5^Global and Tropical Health Division, Menzies School of Health Research and Charles Darwin University, Darwin, Australia

^6^Centre for Epidemiology and Biostatistics, Melbourne School of Population and Global Health, The University of Melbourne, Melbourne, Australia

*Corresponding authors:

**Prabin Dahal**: [prabin.dahal@ndm.ox.ac.uk](mailto:prabin.dahal@ndm.ox.ac.uk)

**Julie A Simpson**: [julieas@unimelb.edu.au](mailto:julieas@unimelb.edu.au)

**Section A: Estimating treatment efficacy for antimalarial drugs**

The primary endpoint in clinical studies of uncomplicated *Plasmodium falciparum* malaria is the occurrence of **recrudescent parasitaemia**, defined as recurrence due to the same parasite, which caused the original infection. Recrudescence occurs when the peripheral parasitaemia initially falls below the level of detection, but subsequently expands after the concentration of the antimalarial drug falls below the minimum inhibitory concentration (Figure 1). Recrudescence is the true treatment failure, since the parasite has evaded complete cure. The current approach for defining antimalarial efficacy is based on the ability of a drug to prevent the subsequent recrudescence^1^. Parasite recurrence can also be due to a heterologous parasite, which can either be a **new infection** with *P. falciparum* or another species of Plasmodia during the ensuing follow-up ^2–4^.

It is important to distinguish recrudescence from new infection so that drug efficacy attributable to parasite drug resistance can be accurately estimated. Recrudescence and new infection remain clinically indistinguishable thus necessitating usage of molecular genotyping technique, Polymerase Chain Reaction (PCR) ^5–7^. The current approach for genotyping uses three polymorphic markers: merozoite surface protein (*msp)-1*, *msp-2,* and glutamate rich protein (*glurp)* genes. If at least one allele at each locus is common in pre- and post-recurrence samples, this is defined as a recrudescence, and when the alleles in the recurrent samples are different from those in the baseline samples, it is considered a new infection ^8^.

When the paired analysis of pre and post treatment parasite genotypes cannot reliably determine the cause of parasitic recurrence, such outcomes are referred as “**indeterminate**”. The current WHO recommendation is to excluded the indeterminate outcomes when deriving antimalarial drug efficacy^1^.


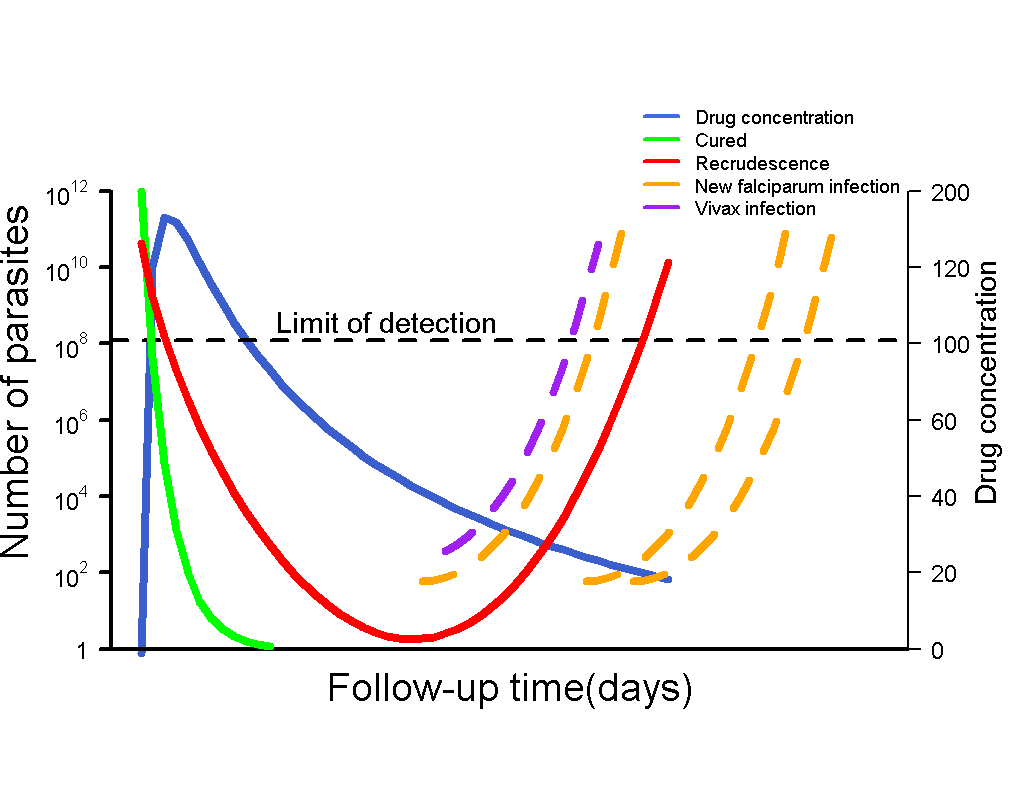


**Figure 1: Therapeutic responses post antimalarial treatment in *P. falciparum* malaria.** Adapted from White NJ: The assessment of antimalarial drug efficacy. *Trends Parasitol* 2002, 18:458–464.^9^

**Legend**: The blue line represents a hypothetical concentration versus time profile for an antimalarial drug administered orally. The green and red lines represent scenarios for parasite burden versus time profiles following treatment for an infection where all the parasites are completely killed resulting in cure (green) and an infection where parasites are initially killed by high drug levels but with drug levels below the minimum inhibitory concentration (MIC), net parasite growth results in subsequent recrudescence (red). The purple and orange lines represent parasite-time profiles for new infections; either an infection due to a new parasite of the same species (orange) or an infection with a *Plasmodium vivax* parasite (purple) during the follow-up. The left y-axis is for parasite density, and the right y-axis shows drug levels at hypothetical units. The horizontal black line represents the microscopic limit of detection for parasites. The maximum number of parasites a human body can contain is 10^12^.

**Table 1: Risk factors associated with missing PCR outcome in the motivating dataset**

|  | PCR outcome missing  No/Yes | Odds Ratio  (95% CI) | *P*-value |
| --- | --- | --- | --- |
| Treatment (reference=AL) | 1160/29 | **-** | **-** |
| ASAQ | 909/20 | 0.85 [0.47-1.53] | 0.585 |
| DP | 1362/13 | 0.39 [0.20-0.76] | 0.006 |
| Transmission (ref=High) | 1687/42 | - | **-** |
| Low | 695/4 | 0.23 [0.08-0.66] | 0.006 |
| Moderate | 1049/16 | 0.70 [0.39-1.28] | 0.249 |

AL = artemether-lumefantrine, ASAQ = artesunate-amodiaquine, DP = dihydroartemisinin-piperaquine

**Table 2: Strength of the association between variables included in the missingness model and PCR adjusted treatment failure in the motivating dataset**

|  | Recrudescence  No/Yes | Odds Ratio  (95% CI) | *P*-value |
| --- | --- | --- | --- |
| Age in years | 3369/81 | 0.84 [0.67-1.04] | 0.108 |
| mg/kg partner dose ^a^ | 3369/81 | 0.73 [0.57-0.94] | 0.013 |
| Time of recurrence | 3369/81 | 0.76 [0.71-0.8] | <0.001 |
| Treatment (ref = AL) | 1131/41 |  |  |
| ASAQ | 889/18 | 0.70 [0.39-1.29] | 0.260 |
| DP | 1349/22 | 0.81 [0.45-1.46] | 0.495 |
| Transmission (ref=High) | 1645/57 |  |  |
| Moderate | 1033/15 | 0.63 [0.34-1.16] | 0.135 |
| Low | 691/9 | 0.63 [0.30-1.31] | 0.215 |

^a^ The mg/kg dose was centred around the mean value for each partner drug

AL = artemether-lumefantrine, ASAQ = artesunate-amodiaquine, DP = dihydroartemisinin-piperaquine ; PCR= Polymerase chain reaction ; CI = Confidence Interval

**Section B:**

**B1: Quantifying bias in complete case estimator**

The following derivation are credited to Prof. Roderick Joseph Little, who generously spent time for these derivations on his peer-reviewed report of the manuscript.

Let, $n$ be the total number of patients who were treated with an antimalarial. We define the following notations:

$$n_{0}=number of cured patients$$

$$m_{1}=number of new infections$$

$$m_{2}=number of recrudescences$$

$$r=number of indeterminate outcomes$$

$$\Rightarrow n=\left( n_{0}+m_{1}+m_{2}+r \right)$$

Under complete case analysis, the estimate of failure proportion is given by:

$$\hat{\rho}_{cc}=\frac{m_{2}}{n_{0}+m_{1}+m_{2}}$$

The bias in complete case estimator can be obtained as:

$$\hat{Bias_{cc}}=\frac{\hat{\pi}.\hat{p}_{0}.\hat{p_{2}}}{\hat{p_{0}}+\left( 1-\hat{\pi} \right).\hat{p}_{2}.(1-\hat{p}_{0})}$$

Where $\hat{\pi}=\frac{r}{(n-n_{0})}$ ; $\hat{p}_{0}=\frac{n_{0}}{n}$; $\hat{p_{1}}=\left( \frac{m_{1}}{m_{1}+m_{2}} \right)$; $\hat{p_{2}}=\left( \frac{m_{1}}{m_{1}+m_{2}} \right)$

The maximum likelihood estimate of the failure proportion, under the multinomial assumption and assuming no covariates is obtained as described by Little and Rubin (2002)^[[1]](#footnote-1)^

$$\hat{\rho}_{ML}=\left( \frac{m_{2}}{m_{1}+m_{2}} \right).\left( \frac{{n-n}_{0}}{n} \right)$$

**B2: Variance of the maximum likelihood estimator**

The Large sample variance of $\hat{\rho}_{MLE}$ can be approximated following equation 13.7 from Little and Rubin (2002)^[[2]](#footnote-2)^

$$Var \left( \hat{\rho}_{ML} \right)\approx\frac{\hat{\rho}_{ML}.\left( 1-\hat{\rho}_{ML} \right)}{\left( n_{0}+m_{1}+m_{2} \right)}\left\{ 1-\left( \frac{\hat{\lambda}{-\hat{\rho}}_{ML}}{1-\hat{\rho}_{ML}} \right).\left( \frac{n-\left( n_{0}+m_{1}+m_{2} \right)}{n} \right)+\left( \left( \frac{{(n}_{0}+m_{1}+m_{2}).t_{+}}{\tau_{+}} \right) -1 \right).\left( \frac{1-\hat{\lambda}}{1-\hat{\rho}_{ML}} \right) \right\}$$

Where $\hat{\lambda}$is the conditional probability of recrudescence given any recurrence among clearly differentiated recurrences, $t_{+}$ is the marginal probability of recurrence among all patients, and $\tau_{+}$ is the overall number of clearly differentiated recurrences estimated respectively as:

$\hat{\lambda}=\frac{m_{2}}{m_{1}+m_{2}}$;$t_{+}=\frac{m_{1}+m_{2}+r}{n}$ ; $\tau_{+}=m_{1}+m_{2}$.

**
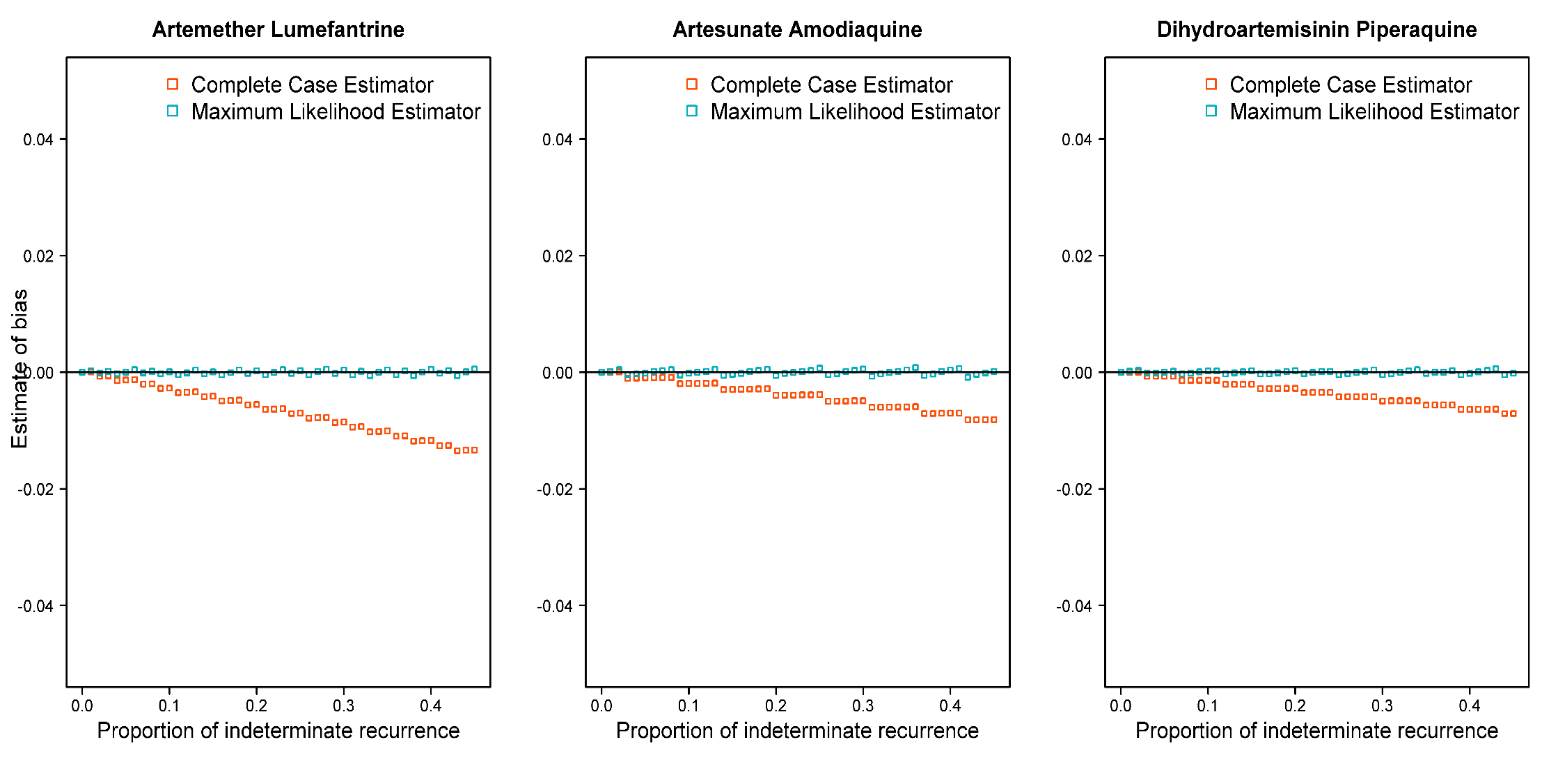
**

**Figure 2: Theoretical estimate of bias for a given proportion of recurrences set as missing (indeterminate) under Missing At Random**

Legend: The maximum likelihood estimate of the failure proportion, under the multinomial assumption and assuming no covariates

**Section C: Application of Rubin’s combination rules for pooling multiply imputed Kaplan-Meier estimates**

With M sets of imputations, let $\hat{S_{m}}$ be the estimate of the Kaplan-Meier (K-M) survival probability for $m^{th}$imputation, m=1,2,3,..,M. The final survival estimate was then obtained by averaging over M imputations as $\bar{S}\left( t \right)=\sum_{m=1}^{M} \frac{\hat{S_{m}}}{M}$. The variance of $\bar{S}\left( t \right)$ was estimated by accounting for within-imputation (W) variability and between-imputation (B) variability

$$Var\left( \bar{S}\left( t \right) \right)=W+\left( 1+\frac{1}{M} \right)B$$

Where $W=\sum_{m=1}^{M} \frac{w_{m}}{M}$; $w_{j}=var(\hat{S_{m}}$ ) and $B=\frac{1}{(M-1)}\sum_{m=1}^{M} (\hat{S_{m}}{-\bar{S(t)})}^{2}$

An estimate of the variance of the *cloglog* transformed K-M was obtained using Taylor’s series approximation given by the following expression (Collet (2015)^[[3]](#footnote-3)^

$Var\left\{ g\left( X \right) \right\}\approx\left\{ \frac{d g\left( X \right)}{d X} \right\}^{2}Var(X)$

The variance of the complementary log transformed survival estimate was then obtained as:

$Var\left\{ \ln\left( -\ln\left( \hat{S}(t) \right) \right) \right\}\approx\left\{ \frac{1}{\ln\left( \hat{S}(t) \right)} \right\}^{2}Var(\ln\left( \hat{S}(t) \right))$

$$\Rightarrow Var\left\{ \ln\left( -\ln\left( \hat{S}(t) \right) \right) \right\}\approx\left\{ \frac{1}{\ln\left( \hat{S}(t) \right)} \right\}^{2}\frac{1}{{\hat{S}(t)}^{2}}. Var\left( \hat{S}(t) \right)$$

**Section D: Comparison of naïve and bootstrapped standard error for inverse probability weighting approach**

The following tables show the standard errors of the complementary log-log transformed Kaplan-Meier estimates derived using IPW approaches for the three treatment regimens. The naïve estimates of standard errors were derived without incorporating uncertainty in the estimated weights. The second column is the estimate of standard error derived as the standard deviation of the Kaplan-Meier estimate across 200 bootstrap samples. The results are averaged across 1,000 simulation runs.

**Table 3: Standard errors of the naïve and bootstrapped IPW estimator presented alongside the model standard error from multiple imputation approach**

|  | **10% missing** | |  | **30% missing** | |  | | **45% missing** | | |  |
| --- | --- | --- | --- | --- | --- | --- | --- | --- | --- | --- | --- |
|  | Naïve IPW | Bootstrap IPW | MI | Naïve IPW | Bootstrap IPW | | MI | Naïve IPW | Bootstrap IPW | MI | |
| **AL** |  |  |  |  |  | |  |  |  |  | |
| M1 | 0.1584 | 0.1656 | 0.1654 | 0.1586 | 0.1861 | | 0.1823 | 0.1593 | 0.2097 | 0.2001 | |
| M2a | 0.1585 | 0.1661 | 0.1651 | 0.1586 | 0.1879 | | 0.1848 | 0.1592 | 0.2123 | 0.2041 | |
| M2b | 0.1585 | 0.1664 | 0.1654 | 0.1587 | 0.1889 | | 0.1857 | 0.1593 | 0.2151 | 0.206 | |
| **ASAQ** |  |  |  |  |  | |  |  |  |  | |
| M1 | 0.2418 | 0.2532 | 0.2505 | 0.2426 | 0.2858 | | 0.2823 | 0.2450 | 0.3230 | 0.3128 | |
| M2a | 0.2417 | 0.2527 | 0.2522 | 0.2425 | 0.2846 | | 0.2802 | 0.2446 | 0.3198 | 0.3090 | |
| M2b | 0.2415 | 0.2520 | 0.2538 | 0.2424 | 0.2826 | | 0.2790 | 0.2443 | 0.3173 | 0.3095 | |
| **DP** |  |  |  |  |  | |  |  |  |  | |
| M1 | 0.2178 | 0.2274 | 0.2247 | 0.2186 | 0.2542 | | 0.2447 | 0.2190 | 0.2841 | 0.2667 | |
| M2a | 0.2177 | 0.2260 | 0.2252 | 0.2182 | 0.2490 | | 0.2441 | 0.2187 | 0.2754 | 0.2609 | |
| M2b | 0.2177 | 0.2246 | 0.2218 | 0.2181 | 0.2444 | | 0.2382 | 0.2187 | 0.2682 | 0.2569 | |

AL= artemether-lumefantrine; ASAQ = artesunate-amodiaquine; DP= dihydroartemisinin-piperaquine; Naïve SEs = SEs for the IPW estimator without incorporating uncertainty in the estimated weights; Bootstrap SEs = SEs derived as the standard deviation of the Kaplan-Meier estimate across 200 bootstrap samples; SE = standard error after complementary log-log (cloglog) transformation of K-M estimate; MI = Multiple Imputation; M1 = Missingness Mechanism 1; M2a = Missingness Mechanism 2a; M2b = Missingness Mechanism 2b

**Section E: Additional results on performance measures for the simulation study**

Table 4: Performance measures of different approaches for handling missing outcome for artemether-lumefantrine [Full data Kaplan-Meier estimate of day 28 cure (SE) = 0.960 (0.1559)]

|  | **10% Missing** | | | | | | **30% missing** | | | | | | **45% missing** | | | | | |
| --- | --- | --- | --- | --- | --- | --- | --- | --- | --- | --- | --- | --- | --- | --- | --- | --- | --- | --- |
|  | Bias | rBias | ModSE† | EmpSE | CP | RMSE | Bias | rBias | ModSE† | EmpSE | CP | RMSE | Bias | rBias | ModSE† | EmpSE | CP | RMSE |
| **M1** |  |  |  |  |  |  |  |  |  |  |  |  |  |  |  |  |  |  |
| CC | 0.0034 | 0.35 | 0.1672 | 0.1505 | 0.909 | 0.1815 | 0.0104 | 1.08 | 0.1905 | 0.1774 | 0.504 | 0.3691 | 0.0159 | 1.65 | 0.2152 | 0.2032 | 0.134 | 0.5732 |
| IPW | 0.0002 | 0.02 | 0.1656 | 0.1543 | 0.958 | 0.1551 | 0.0001 | 0.01 | 0.1861 | 0.1742 | 0.957 | 0.1749 | 0.0002 | 0.02 | 0.2097 | 0.2015 | 0.954 | 0.2028 |
| MI | -0.0002 | -0.02 | 0.1654 | 0.1600 | 0.962 | 0.1601 | -0.0012 | -0.13 | 0.1823 | 0.1707 | 0.961 | 0.1715 | -0.0026 | -0.27 | 0.2001 | 0.1854 | 0.944 | 0.1914 |
| IPW-E | 0.0018 | 0.19 | 0.1660 | 0.1544 | 0.946 | 0.1652 | 0.0039 | 0.40 | 0.1968 | 0.1867 | 0.903 | 0.2224 | 0.0039 | 0.41 | 0.2536 | 0.2582 | 0.851 | 0.2918 |
| **M2a** |  |  |  |  |  |  |  |  |  |  |  |  |  |  |  |  |  |  |
| CC | 0.0035 | 0.37 | 0.1676 | 0.1511 | 0.905 | 0.1840 | 0.0108 | 1.12 | 0.1920 | 0.1788 | 0.471 | 0.3809 | 0.0163 | 1.69 | 0.2176 | 0.2053 | 0.123 | 0.5907 |
| IPW | 0.0002 | 0.02 | 0.1661 | 0.1566 | 0.96 | 0.1574 | 0.0001 | 0.01 | 0.1879 | 0.1769 | 0.957 | 0.1776 | 0.0001 | 0.01 | 0.2123 | 0.2028 | 0.957 | 0.2039 |
| MI | -0.0003 | -0.04 | 0.1651 | 0.1535 | 0.966 | 0.1535 | -0.0012 | -0.12 | 0.1848 | 0.1846 | 0.947 | 0.1850 | -0.0022 | -0.23 | 0.2041 | 0.1901 | 0.955 | 0.1938 |
| IPW-E | 0.0018 | 0.19 | 0.1664 | 0.1571 | 0.947 | 0.1681 | 0.0040 | 0.42 | 0.2001 | 0.1910 | 0.893 | 0.2281 | 0.0039 | 0.41 | 0.2594 | 0.2680 | 0.837 | 0.3019 |
| **M2b** |  |  |  |  |  |  |  |  |  |  |  |  |  |  |  |  |  |  |
| CC | 0.0036 | 0.38 | 0.1679 | 0.1518 | 0.902 | 0.1861 | 0.0110 | 1.14 | 0.1928 | 0.1800 | 0.458 | 0.3874 | 0.0167 | 1.74 | 0.2200 | 0.2060 | 0.101 | 0.6085 |
| IPW | 0.0002 | 0.02 | 0.1664 | 0.1563 | 0.961 | 0.1570 | 0.0001 | 0.01 | 0.1889 | 0.1787 | 0.961 | 0.1794 | 0.0001 | 0.01 | 0.2151 | 0.2051 | 0.957 | 0.2063 |
| MI | -0.0005 | -0.05 | 0.1654 | 0.1561 | 0.964 | 0.1560 | -0.0011 | -0.11 | 0.1857 | 0.1741 | 0.954 | 0.1745 | -0.0026 | -0.27 | 0.2060 | 0.1978 | 0.945 | 0.2031 |
| IPW-E | 0.0019 | 0.19 | 0.1668 | 0.1568 | 0.946 | 0.1680 | 0.0040 | 0.42 | 0.2016 | 0.1927 | 0.894 | 0.2304 | 0.0040 | 0.41 | 0.2659 | 0.2772 | 0.831 | 0.3119 |

†The model based standard errors for IPW approaches were estimated using 200 bootstrap samples

M1 = Missingness Mechanism 1; M2a = Missingness Mechanism 2a; M2b = Missingness Mechanism 2b; rBias = Relative bias (%); ModSE = Model based standard error; EmpSE = Empirical standard error; CP = Coverage Probability; RMSE = Root Mean Squared Error; CC = Complete Case; IPW = Inverse probability weighting model; MI = Multiply Imputed; IPW E = IPW model which excluded recurrence as a predictor in missingness model

SE, ModSE and EmpSE are presented on complementary log-log (cloglog) scale

Table 5: Performance measures of different approaches for handling missing outcome for artesunate-amodiaquine [Full data Kaplan-Meier estimate of day 28 cure (SE) = 0.979 (0.2367)]

|  | **10% Missing** | | | | | | **30% missing** | | | | | | **45% missing** | | | | | |
| --- | --- | --- | --- | --- | --- | --- | --- | --- | --- | --- | --- | --- | --- | --- | --- | --- | --- | --- |
|  | Bias | rBias | ModSE† | EmpSE | CP | RMSE | Bias | rBias | ModSE† | EmpSE | CP | RMSE | Bias | rBias | ModSE† | EmpSE | CP | RMSE |
| **M1** |  |  |  |  |  |  |  |  |  |  |  |  |  |  |  |  |  |  |
| CC | 0.0018 | 0.26 | 0.2560 | 0.2438 | 0.896 | 0.2716 | 0.0056 | 0.57 | 0.2922 | 0.2787 | 0.720 | 0.4469 | 0.0086 | 0.88 | 0.3329 | 0.3166 | 0.407 | 0.6594 |
| IPW | 0.0001 | 0.25 | 0.2532 | 0.2499 | 0.941 | 0.2519 | -0.0001 | -0.01 | 0.2858 | 0.2850 | 0.940 | 0.2870 | 0.0000 | 0.00 | 0.3230 | 0.3249 | 0.934 | 0.3284 |
| MI | -0.0006 | 0.25 | 0.2505 | 0.2326 | 0.958 | 0.2325 | -0.0012 | -0.13 | 0.2823 | 0.2639 | 0.955 | 0.2649 | -0.0021 | -0.22 | 0.3128 | 0.2878 | 0.947 | 0.2937 |
| IPW-E | 0.0012 | 0.25 | 0.2546 | 0.2505 | 0.926 | 0.2647 | 0.0029 | 0.30 | 0.2942 | 0.2921 | 0.885 | 0.3470 | 0.0038 | 0.39 | 0.3496 | 0.3579 | 0.818 | 0.4405 |
| **M2a** |  |  |  |  |  |  |  |  |  |  |  |  |  |  |  |  |  |  |
| CC | 0.0018 | 0.26 | 0.2556 | 0.2442 | 0.897 | 0.2706 | 0.0055 | 0.56 | 0.2906 | 0.2774 | 0.725 | 0.4383 | 0.0085 | 0.86 | 0.3301 | 0.3154 | 0.437 | 0.6455 |
| IPW | 0.0001 | 0.25 | 0.2527 | 0.2490 | 0.946 | 0.2509 | -0.0001 | -0.01 | 0.2846 | 0.2827 | 0.942 | 0.2846 | -0.0001 | -0.01 | 0.3198 | 0.3241 | 0.931 | 0.3270 |
| MI | -0.0003 | 0.25 | 0.2522 | 0.2418 | 0.959 | 0.2422 | -0.0014 | -0.15 | 0.2802 | 0.2683 | 0.954 | 0.2702 | -0.0025 | -0.25 | 0.3090 | 0.2929 | 0.934 | 0.3017 |
| IPW-E | 0.0011 | 0.25 | 0.2540 | 0.2492 | 0.928 | 0.2632 | 0.0029 | 0.29 | 0.2924 | 0.2899 | 0.886 | 0.3437 | 0.0037 | 0.37 | 0.3447 | 0.3536 | 0.833 | 0.4323 |
| **M2b** |  |  |  |  |  |  |  |  |  |  |  |  |  |  |  |  |  |  |
| CC | 0.0017 | 0.25 | 0.2548 | 0.2437 | 0.902 | 0.2679 | 0.0052 | 0.53 | 0.2877 | 0.2733 | 0.736 | 0.4224 | 0.0082 | 0.84 | 0.3267 | 0.3125 | 0.459 | 0.6277 |
| IPW | 0.0001 | 0.25 | 0.2520 | 0.2457 | 0.946 | 0.2475 | -0.0001 | -0.01 | 0.2826 | 0.2804 | 0.941 | 0.2822 | -0.0001 | -0.01 | 0.3173 | 0.3199 | 0.936 | 0.3226 |
| MI | 0.0000 | 0.25 | 0.2538 | 0.2467 | 0.965 | 0.2480 | -0.0011 | -0.12 | 0.2790 | 0.2533 | 0.965 | 0.2542 | -0.0021 | -0.21 | 0.3095 | 0.2870 | 0.959 | 0.2923 |
| IPW-E | 0.0011 | 0.25 | 0.2532 | 0.2459 | 0.930 | 0.2591 | 0.0028 | 0.28 | 0.2892 | 0.2868 | 0.891 | 0.3385 | 0.0036 | 0.37 | 0.3397 | 0.3457 | 0.837 | 0.4219 |

†The model based standard errors for IPW approaches were estimated using 200 bootstrap samples

M1 = Missingness Mechanism 1; M2a = Missingness Mechanism 2a; M2b = Missingness Mechanism 2b; rBias = Relative bias (%); ModSE = Model based standard error; EmpSE = Empirical standard error; CP = Coverage Probability; RMSE = Root Mean Squared Error; CC = Complete Case; IPW = Inverse probability weighting model; MI = Multiply Imputed; IPW E = IPW model which excluded recurrence as a predictor in missingness model

SE, ModSE and EmpSE are presented on complementary log-log (cloglog) scale

Table 6: Performance measures of different approaches for handling missing outcome for dihydroartemisinin-piperaquine [Full data Kaplan-Meier estimate of day 28 cure (SE) = 0.983 (0.2082)]

|  | **10% Missing** | | | | | | **30% missing** | | | | | | **45% missing** | | | | | |
| --- | --- | --- | --- | --- | --- | --- | --- | --- | --- | --- | --- | --- | --- | --- | --- | --- | --- | --- |
|  | Bias | rBias | ModSE† | EmpSE | CP | RMSE | Bias | rBias | ModSE† | EmpSE | CP | RMSE | Bias | rBias | ModSE† | EmpSE | CP | RMSE |
| **M1** |  |  |  |  |  |  |  |  |  |  |  |  |  |  |  |  |  |  |
| CC | 0.0016 | 0.16 | 0.2296 | 0.2071 | 0.904 | 0.2391 | 0.0047 | 0.48 | 0.2618 | 0.2385 | 0.605 | 0.4334 | 0.0076 | 0.73 | 0.2997 | 0.2903 | 0.256 | 0.6726 |
| IPW | 0.0001 | 0.01 | 0.2274 | 0.2158 | 0.957 | 0.2179 | 0.0001 | 0.01 | 0.2542 | 0.2470 | 0.95 | 0.2493 | 0.0044 | -0.01 | 0.2841 | 0.2812 | 0.946 | 0.2831 |
| MI | -0.0003 | -0.03 | 0.2247 | 0.2131 | 0.961 | 0.2131 | -0.0010 | -0.10 | 0.2447 | 0.2234 | 0.957 | 0.2257 | 0.0045 | -0.15 | 0.2667 | 0.2457 | 0.948 | 0.2528 |
| IPW-E | 0.0014 | 0.14 | 0.2298 | 0.2156 | 0.923 | 0.2415 | 0.0036 | 0.37 | 0.2703 | 0.2635 | 0.766 | 0.3851 | 0.0062 | 0.49 | 0.3342 | 0.3490 | 0.601 | 0.5332 |
| **M2a** |  |  |  |  |  |  |  |  |  |  |  |  |  |  |  |  |  |  |
| CC | 0.0013 | 0.14 | 0.2276 | 0.2042 | 0.917 | 0.2292 | 0.0042 | 0.43 | 0.2562 | 0.2340 | 0.673 | 0.3979 | 0.0070 | 0.66 | 0.2882 | 0.2755 | 0.337 | 0.6028 |
| IPW | 0.0002 | 0.02 | 0.2260 | 0.2120 | 0.955 | 0.2141 | 0.0001 | 0.01 | 0.2490 | 0.2397 | 0.951 | 0.2416 | 0.0043 | 0.00 | 0.2754 | 0.2737 | 0.943 | 0.2754 |
| MI | 0.0000 | 0.00 | 0.2252 | 0.2064 | 0.972 | 0.2073 | -0.0005 | -0.05 | 0.2441 | 0.2254 | 0.971 | 0.2253 | 0.0042 | -0.13 | 0.2609 | 0.2381 | 0.953 | 0.2432 |
| IPW-E | 0.0012 | 0.12 | 0.2281 | 0.2121 | 0.927 | 0.2335 | 0.0032 | 0.32 | 0.2626 | 0.2528 | 0.804 | 0.3521 | 0.0058 | 0.43 | 0.3208 | 0.3353 | 0.653 | 0.4856 |
| **M2b** |  |  |  |  |  |  |  |  |  |  |  |  |  |  |  |  |  |  |
| CC | 0.0011 | 0.11 | 0.2260 | 0.2025 | 0.928 | 0.2217 | 0.0037 | 0.38 | 0.2506 | 0.2306 | 0.733 | 0.3632 | 0.0064 | 0.60 | 0.2782 | 0.2598 | 0.424 | 0.5397 |
| IPW | 0.0002 | 0.02 | 0.2246 | 0.2108 | 0.959 | 0.2128 | 0.0001 | 0.01 | 0.2444 | 0.2354 | 0.951 | 0.2374 | 0.0041 | 0.00 | 0.2682 | 0.2643 | 0.946 | 0.2662 |
| MI | -0.0003 | -0.03 | 0.2218 | 0.2010 | 0.974 | 0.2009 | -0.0006 | -0.06 | 0.2382 | 0.2128 | 0.972 | 0.2130 | 0.0041 | -0.10 | 0.2569 | 0.2380 | 0.960 | 0.2401 |
| IPW-E | 0.0010 | 0.11 | 0.2263 | 0.2103 | 0.933 | 0.2275 | 0.0028 | 0.28 | 0.2555 | 0.2462 | 0.830 | 0.3254 | 0.0054 | 0.38 | 0.3082 | 0.3178 | 0.709 | 0.4418 |

†The model based standard errors for IPW approaches were estimated using 200 bootstrap samples

M1 = Missingness Mechanism 1; M2a = Missingness Mechanism 2a; M2b = Missingness Mechanism 2b; rBias = Relative bias (%); ModSE = Model based standard error; EmpSE = Empirical standard error; CP = Coverage Probability; RMSE = Root Mean Squared Error; CC = Complete Case; IPW = Inverse probability weighting model; MI = Multiply Imputed; IPW E = IPW model which excluded recurrence as a predictor in missingness model

SE, ModSE and EmpSE are presented on complementary log-log (cloglog) scale

Table 7: Performance measures of different approaches for handling missing outcome for AL for estimating cured proportion [Full data estimate of day 28 cured proportion (SE) = 0.964(0.1562)]

|  | **10% Missing** | | | | | | **30% missing** | | | | | | **45% missing** | | | | | |
| --- | --- | --- | --- | --- | --- | --- | --- | --- | --- | --- | --- | --- | --- | --- | --- | --- | --- | --- |
| **M1** | Bias | rBias | ModSE† | EmpSE | CP | RMSE | Bias | rBias | ModSE† | EmpSE | CP | RMSE | Bias | rBias | ModSE† | EmpSE | CP | RMSE |
| CC | 0.2643 | 0.27 | 0.1664 | 0.1627 | 0.908 | 0.1857 | 0.8692 | 0.90 | 0.1897 | 0.1935 | 0.5770 | 0.3539 | 1.3724 | 1.42 | 0.2153 | 0.2177 | 0.2140 | 0.5503 |
| MLE | -0.0002 | -0.02 | 0.1655 | 0.1631 | 0.943 | 0.1632 | -0.0001 | -0.01 | 0.1866 | 0.1925 | 0.9350 | 0.1929 | 0.0000 | 0.00 | 0.2100 | 0.2159 | 0.9310 | 0.2169 |
| **M2a** |  |  |  |  |  |  |  |  |  |  |  |  |  |  |  |  |  |  |
| CC | 0.2748 | 0.29 | 0.1668 | 0.1637 | 0.909 | 0.1882 | 0.9014 | 0.94 | 0.1914 | 0.1977 | 0.5410 | 0.3668 | 1.4093 | 1.46 | 0.2179 | 0.2227 | 0.1920 | 0.5686 |
| MLE | -0.0003 | -0.03 | 0.1659 | 0.1636 | 0.944 | 0.1636 | -0.0004 | -0.04 | 0.1881 | 0.1954 | 0.9330 | 0.1954 | -0.0004 | -0.04 | 0.2123 | 0.2185 | 0.9340 | 0.2186 |
| **M2b** |  |  |  |  |  |  |  |  |  |  |  |  |  |  |  |  |  |  |
| CC | 0.2811 | 0.29 | 0.1671 | 0.1633 | 0.901 | 0.1888 | 0.9190 | 0.95 | 0.1923 | 0.1973 | 0.5300 | 0.3721 | 1.4437 | 1.50 | 0.2202 | 0.2248 | 0.1720 | 0.5844 |
| MLE | -0.0004 | -0.04 | 0.1661 | 0.1632 | 0.948 | 0.1631 | -0.0006 | -0.07 | 0.1888 | 0.1941 | 0.9350 | 0.1940 | -0.0009 | -0.09 | 0.2143 | 0.2204 | 0.9380 | 0.2203 |

M1 = Missingness Mechanism 1; M2a = Missingness Mechanism 2a; M2b = Missingness Mechanism 2b; rBias = Relative bias (%); ModSE = Model based standard error; EmpSE = Empirical standard error; CP = Coverage Probability; RMSE = Root Mean Squared Error; CC = Complete Case; MLE = Maximum likelihood estimator of cured proportion

SE, ModSE and EmpSE are presented on complementary log-log (cloglog) scale

Table 8: Performance measures of different approaches for handling missing outcome for ASAQ for estimating cured proportion [Full data estimate of day 28 cured proportion (SE) = 0.980(0.2357)]

|  | **10% Missing** | | | | | | **30% missing** | | | | | | **45% missing** | | | | | |
| --- | --- | --- | --- | --- | --- | --- | --- | --- | --- | --- | --- | --- | --- | --- | --- | --- | --- | --- |
| **M1** | Bias | rBias | ModSE† | EmpSE | CP | RMSE | Bias | rBias | ModSE† | EmpSE | CP | RMSE | Bias | rBias | ModSE† | EmpSE | CP | RMSE |
| CC | 0.1668 | 0.17 | 0.2563 | 0.2562 | 0.882 | 0.2817 | 0.5373 | 0.55 | 0.2951 | 0.3009 | 0.722 | 0.4635 | 0.8329 | 0.85 | 0.3393 | 0.3500 | 0.399 | 0.6862 |
| MLE | -0.0001 | -0.01 | 0.2550 | 0.2560 | 0.944 | 0.2573 | -0.0001 | -0.01 | 0.2907 | 0.3005 | 0.930 | 0.3028 | 0.0001 | 0.01 | 0.3320 | 0.3498 | 0.909 | 0.3544 |
| **M2a** |  |  |  |  |  |  |  |  |  |  |  |  |  |  |  |  |  |  |
| CC | 0.1591 | 0.16 | 0.2557 | 0.2553 | 0.885 | 0.2791 | 0.5198 | 0.53 | 0.2933 | 0.3008 | 0.733 | 0.4545 | 0.8105 | 0.83 | 0.3358 | 0.3472 | 0.423 | 0.6679 |
| MLE | -0.0001 | -0.01 | 0.2544 | 0.2548 | 0.939 | 0.2558 | -0.0002 | -0.02 | 0.2889 | 0.2985 | 0.928 | 0.2998 | -0.0002 | -0.02 | 0.3285 | 0.3450 | 0.922 | 0.3478 |
| **M2b** |  |  |  |  |  |  |  |  |  |  |  |  |  |  |  |  |  |  |
| CC | 0.1499 | 0.15 | 0.2549 | 0.2543 | 0.889 | 0.2761 | 0.4958 | 0.51 | 0.2907 | 0.3003 | 0.745 | 0.4422 | 0.7867 | 0.80 | 0.3321 | 0.3450 | 0.447 | 0.6495 |
| MLE | -0.0002 | -0.02 | 0.2537 | 0.2539 | 0.941 | 0.2546 | -0.0003 | -0.03 | 0.2865 | 0.2983 | 0.925 | 0.2991 | -0.0004 | -0.04 | 0.3249 | 0.3422 | 0.922 | 0.3436 |

M1 = Missingness Mechanism 1; M2a = Missingness Mechanism 2a; M2b = Missingness Mechanism 2b; rBias = Relative bias (%); ModSE = Model based standard error; EmpSE = Empirical standard error; CP = Coverage Probability; RMSE = Root Mean Squared Error; CC = Complete Case; MLE = Maximum likelihood estimator of cured proportion

SE, ModSE and EmpSE are presented on complementary log-log (cloglog) scale

Table 9: Performance measures of different approaches for handling missing outcome for DP for estimating cured proportion [Full data estimate of day 28 cured proportion (SE) = 0.984 (0.2132)]

|  | **10% Missing** | | | | | | **30% missing** | | | | | | | **45% missing** | | | | | | |
| --- | --- | --- | --- | --- | --- | --- | --- | --- | --- | --- | --- | --- | --- | --- | --- | --- | --- | --- | --- | --- |
| **M1** | Bias | rBias | ModSE† | EmpSE | CP | RMSE | Bias | rBias | ModSE† | EmpSE | CP | RMSE | Bias | | rBias | ModSE† | EmpSE | CP | RMSE |  |
| CC | 0.1630 | 0.17 | 0.2304 | 0.2051 | 0.907 | 0.2408 | 0.4718 | 0.48 | 0.2634 | 0.2410 | 0.577 | 0.4427 | 0.7094 | | 0.721 | 0.3003 | 0.2789 | 0.276 | 0.6712 |  |
| MLE | 0.0001 | 0.01 | 0.2282 | 0.2049 | 0.962 | 0.2066 | 0.0001 | 0.01 | 0.2562 | 0.2376 | 0.950 | 0.2402 | 0.0002 | | 0.020 | 0.2881 | 0.2715 | 0.947 | 0.2753 |  |
| **M2a** |  |  |  |  |  |  |  |  |  |  |  |  |  | |  |  |  |  |  |  |
| CC | 0.1411 | 0.14 | 0.2285 | 0.2036 | 0.919 | 0.2319 | 0.4250 | 0.43 | 0.2576 | 0.2352 | 0.640 | 0.4054 | 0.6502 | | 0.661 | 0.2901 | 0.2721 | 0.344 | 0.6104 |  |
| MLE | 0.0001 | 0.01 | 0.2266 | 0.2028 | 0.962 | 0.2042 | 0.0001 | 0.01 | 0.2512 | 0.2318 | 0.955 | 0.2340 | 0.0002 | | 0.016 | 0.2792 | 0.2645 | 0.951 | 0.2677 |  |
| **M2b** |  |  |  |  |  |  |  |  |  |  |  |  |  | |  |  |  |  |  |  |
| CC | 0.1193 | 0.12 | 0.2267 | 0.2027 | 0.926 | 0.2244 | 0.3739 | 0.38 | 0.2518 | 0.2316 | 0.708 | 0.3693 | 0.5908 | | 0.601 | 0.2802 | 0.2584 | 0.407 | 0.5489 |  |
| MLE | 0.0000 | 0.00 | 0.2251 | 0.2021 | 0.959 | 0.2032 | 0.0001 | 0.01 | 0.2462 | 0.2285 | 0.955 | 0.2303 | 0.0001 | | 0.007 | 0.2704 | 0.2524 | 0.957 | 0.2546 |  |

M1 = Missingness Mechanism 1; M2a = Missingness Mechanism 2a; M2b = Missingness Mechanism 2b; rBias = Relative bias (%); ModSE = Model based standard error; EmpSE = Empirical standard error; CP = Coverage Probability; RMSE = Root Mean Squared Error; CC = Complete Case; MLE = Maximum likelihood estimator of cured proportion

SE, ModSE and EmpSE are presented on complementary log-log (cloglog) scale

**References**

1. World Health Organization. *Methods for Surveillance of Antimalarial Drug Efficacy*. (2009).

2. Prentice, R. L. *et al.* The analysis of failure times in the presence of competing risks. *Biometrics* **34**, 541–54 (1978).

3. Putter, H., Fiocco, M. & Geskus, R. B. Tutorial in biostatistics: Competing risks and multi-state models. *Stat. Med.* **26**, 4817–4834 (2007).

4. Wolbers, M. *et al.* Competing risks analyses: objectives and approaches. *Eur. Heart J.* **35**, 2936–2941. (2014).

5. Snounou, G. & Beck, H. P. The use of PCR genotyping in the assessment of recrudescence or reinfection after antimalarial drug treatment. *Parasitol. Today* **14**, 462–467 (1998).

6. Brockman, A. *et al.* Application of genetic markers to the identification of recrudescent Plasmodium falciparum infections on the northwestern border of Thailand. *Am J Trop Med Hyg* **60**, 14–21 (1999).

7. Juliano, J. J., Gadalla, N., Sutherland, C. J. & Meshnick, S. R. The perils of PCR: can we accurately ‘correct’ antimalarial trials? *Trends Parasitol.* **26**, 119–124 (2010).

8. World Health Organization. *Methods and techniques for clinical trials on antimalarial drug efficacy : genotyping to identify parasite populations*. (2007).

9. White, N. J. The assessment of antimalarial drug efficacy. *Trends Parasitol* **18**, 458–464 (2002).

1. Little and Rubin: Chapter 13: **Models for Partially Classified Contigency Tables, Ignoring the Missing-Data Mechanism**,In Statistical Analysis with Missing Data (2002) [↑](#footnote-ref-1)
2. Little and Rubin: Chapter 13: **Models for Partially Classified Contigency Tables, Ignoring the Missing-Data Mechanism**,In Statistical Analysis with Missing Data (2002) [↑](#footnote-ref-2)
3. Collett D: *Modelling Survival Data in Medical Research, Third Edition*.*p26 (Equation 2.8)*. (2015) [↑](#footnote-ref-3)
